# Minimization of energy transduction confers resistance to phosphine in the rice weevil, *Sitophilus oryzae*

**DOI:** 10.1038/s41598-019-50972-w

**Published:** 2019-10-10

**Authors:** Kyeongnam Kim, Jeong Oh Yang, Jae-Yoon Sung, Ji-Young Lee, Jeong Sun Park, Heung-Sik Lee, Byung-Ho Lee, Yonglin Ren, Dong-Woo Lee, Sung-Eun Lee

**Affiliations:** 1 School of Applied Biosciences, Kyungpook National University, Daegu, 41566 Korea; 2 Animal and Plant Quarantine Agency (APQA), Gimcheon, 39660 Korea; 3 Department of Biotechnology, Yonsei University, Seoul, 03722 Korea; 4 Institute of Agriculture and Life Science, Gyeongsang National University, Jinju, 52828 Korea; 5 School of Veterinary and Life Science, Murdoch University, 90 South St., Murdoch, WA 6150 Australia

**Keywords:** Mechanism of action, Environmental impact

## Abstract

Infestation of phosphine (PH_3_) resistant insects threatens global grain reserves. PH_3_ fumigation controls rice weevil (*Sitophilus oryzae*) but not highly resistant insect pests. Here, we investigated naturally occurring strains of *S. oryzae* that were moderately resistant (MR), strongly resistant (SR), or susceptible (wild-type; WT) to PH_3_ using global proteome analysis and mitochondrial DNA sequencing. Both PH_3_ resistant (PH_3_–R) strains exhibited higher susceptibility to ethyl formate-mediated inhibition of cytochrome *c* oxidase than the WT strain, whereas the disinfectant PH_3_ concentration time of the SR strain was much longer than that of the MR strain. Unlike the MR strain, which showed altered expression levels of genes encoding metabolic enzymes involved in catabolic pathways that minimize metabolic burden, the SR strain showed changes in the mitochondrial respiratory chain. Our results suggest that the acquisition of strong PH_3_ resistance necessitates the avoidance of oxidative phosphorylation through the accumulation of a few non-synonymous mutations in mitochondrial genes encoding complex I subunits as well as nuclear genes encoding dihydrolipoamide dehydrogenase, concomitant with metabolic reprogramming, a recognized hallmark of cancer metabolism. Taken together, our data suggest that reprogrammed metabolism represents a survival strategy of SR insect pests for the compensation of minimized energy transduction under anoxic conditions. Therefore, understanding the resistance mechanism of PH_3_–R strains will support the development of new strategies to control insect pests.

## Introduction

To control insect pests of stored agricultural products, phosphine (PH_3_) has been widely used for fumigation as an alternative to methyl bromide, which depletes the ozone layer in the atmosphere^1^. However, frequent worldwide emergence of PH_3_ resistant (PH_3_–R) insect pests including rice weevil (*Sitophilus oryzae*)^2^, *Cryptolestes ferrugineus*^3,4^, *Rhyzopertha dominica*^5^, and *Tribolium castaneum*^6–8^ in stored grains, because of intensive PH_3_ use and global warming, threatens global grain reserves^9^. Although the frequency of PH_3_–R insects in the field is low, weak PH_3_ resistance has been observed in *S. oryzae*, which may be ascribed to a major autosomal and incompletely recessive gene, without any evidence of a fitness cost in the absence of PH_3_ selection^2^. Recently, PH_3_–R insects have been frequently reported in several countries, including China, Vietnam, Bangladesh, Brazil, Turkey, the United States, Korea, and Australia^4,5,7,8,10–15^, and several PH_3_–R strains of *S. oryzae* have been identified in Australia and Brazil^2,16^ as well as in Korea^17^.

PH_3_–R insect pests harbor an identical amino acid substitution in a core metabolic gene encoding dihydrolipoamide dehydrogenase (DLD), consistent with the presence of a genetic resistance factor identified in *R. dominica* and *T. castaneum*^7^. Variants of DLD represent a PH_3_ resistance factor because metabolite profiles of resistant insect pests differ from those of susceptible pests under PH_3_ exposure^18^. A fitness cost associated with the strongly resistant (SR) allele of the *dld* gene appears to be segregating in insect populations in the absence of selection, implying that the prevalence of resistance is a potential threat to resistant species under considerable selection pressure^11^. Nevertheless, the genetic and physiological mechanisms of PH_3_ resistance in insect pests remain unclear. Understanding the PH_3_ resistance mechanism in PH_3_–R strains is urgently needed not only to screen new pesticides but also to develop a more biologically safe and sustainable method of controlling the global population of pesticide resistant pests of stored agricultural products^19,20^.

Intriguingly, the emergence of strong PH_3_ resistance in *R. dominica* and *T. castaneum* in India, the United States, and Australia, presumably because of the substitution of proline with serine at amino acid position 49 or 45 (P49S or P45S, respectively) in DLD, may be ascribed to a strong selection by the excessive use of PH_3_ fumigation^7,10^. On the other hand, we previously showed that the expression of several key metabolic genes, including those encoding glyceraldehyde-3-phosphate dehydrogenase (GAPDH), triosephosphate isomerase (TPI), and DLD was significantly reduced in the PH_3_–R *R. dominica* strain (CRD343) compared with the PH_3_ susceptible *R. dominica* strain^21^. In addition, genes encoding sodium channel proteins, glutamate racemase, enolase, and vitellogenin were highly expressed in the PH_3_–R strain^21^. These findings may be ascribed to a possible trade-off between survival and proliferation plays a key role in the acquisition of pesticide resistance. However, this result does not support the previous observation that PH_3_ resistance is highly species-specific^21^. Recently, expression profiling of four mitochondrial genes, including *cox1, nad3*, *atp6*, and *cob*, in *C. ferrugineus* by quantitative real-time PCR (qRT-PCR) revealed a strong negative correlation between PH_3_ resistance and respiratory chain function^3^, suggesting that the emergence of fumigant resistance is associated with the modulation of energy production in mitochondria.

In this study, we investigated differences in protein profiles of the susceptible strain and two distinct PH_3_–R strains of *S. oryzae* by performing comparative proteome analysis and ethyl formate (EF) inhibition kinetics. In addition, expression levels of several putative PH_3_–R marker genes were validated by qRT-PCR. Subsequently, we aimed to identify single nucleotide polymorphisms (SNPs) or mutations in complex I (ND) subunit encoding genes through mitochondrial DNA (mtDNA) sequencing of both PH_3_–R *S. oryzae* strains.

## Results

### Differential acute PH_3_ toxicity of the susceptible and resistant *S. oryzae* strains

To assess the extent of PH_3_ resistance in *S. oryzae*, a susceptible wild-type (WT) strain, obtained from Australia, was compared with two resistant strains (R1 and R2) obtained from two geographically distinct Provinces in Korea **(**Table 1**)**. The disinfectant concentration-time (Ct) values (mg·h/L) of the WT and PH_3_–R strains demonstrated that despite the similar level of PH_3_ toxicity (Ct_50_, Ct value to achieve 50% mortality) between WT and R1 strains, the Ct_99_ (Ct value to achieve 99% mortality) value of the R2 strain at 20 °C was 29- and 10-fold higher than that of WT and PH_3_–R1 strains, respectively. In addition, the R2 strain was more resistant to PH_3_ than the R1 and WT strains, presumably because of a mutation in the *dld* gene encoding DLD (Table 1 and Supplementary Table S1). Thus, acute PH_3_ toxicity data indicate that the PH_3_ resistance mechanism of the R2 strain is considerably different from that of the R1 strain. Therefore, to discriminate between these strains, R1 and R2, hereafter we refer to them as moderately resistant (MR) and strongly resistant (SR) *S. oryzae* strains, respectively.

**Table 1:** Analysis of phosphine (PH_3_) resistance in susceptible wild-type (WT) and resistance strains (R1 and R2).

Feature	WT	R1	R2
Country of origin	Perth, Australia	Gunsan, Korea	Cheongju, Korea
Acute toxicity assay^a^			
Ct_50_	0.681 (0.500–0.840)	0.616 (0.164–1.469)	27.053 (21.158–33.020)
Ct_99_	2.930 (2.230–4.604)	8.114 (2.457–135.097)	86.137 (58.991–230.177)
Slope ± SE	3.671 ± 0.442	2.077 ± 0.389	4.626 ± 0.681
df	10	6	7
χ^2^	6.55	0.03	8.76
FAO test^b^	Susceptible	Weakly resistant	Strongly resistant
SNP in DLD^c^	None	None	Yes
Degree of PH_3_ resistance	Susceptible	Moderately resistant	Strongly resistant

^a^Concentration-time (Ct_50_ and Ct_99_) values indicate 50% and 99.9% mortality using a Probit model^43^ at various PH_3_ concentrations (0.01 to 1.0 mg/L) under fumigation conditions for 20 h at 20 °C. Ct values are expressed as concentration × time values (mg h/L). All acute toxicity assays were performed using 30 insects in triplicate. SE, standard error; df, degree of freedom; *χ*^2^ = chi-square.

^b^FAO method No. 16 for testing phosphine resistance^50^

^c^SNP, single nucleotide polymorphism; SNP detection in the *dld* gene encoding DLD was as described by Nguyen *et al*. (see also Supplementary Table S1)^11^.

**Figure 1:**
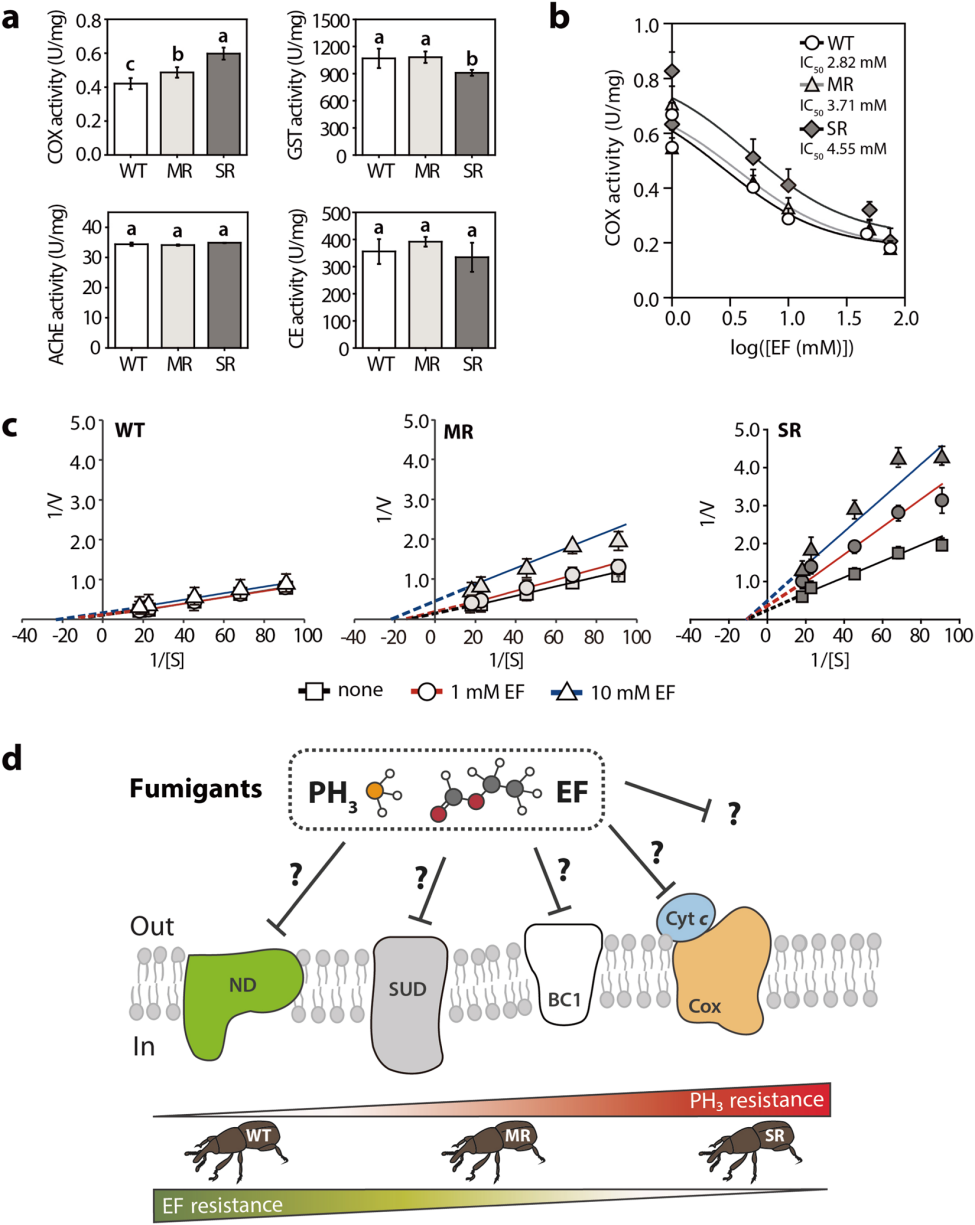
Effect of ethyl formate (EF) on cytochrome *c* oxidase (COX) activity in phosphine (PH_3_) susceptible and resistant strains. (**a**) Enzyme activities of COX, acetylcholinesterase (AChE), glutathione *S*-transferase (GST), carboxylesterase (CE), expressed as unit/mg protein of rice weevils. Significant differences among PH_3_ susceptible (wild-type [WT]) and resistant strains (moderately resistant [MR] and strongly resistant [SR]) were determined using one-way ANOVA (*p* < 0.05), followed by Tukey’s post-hoc tests and are indicated using different letters. (**b**) Dose-response curves constructed by non-linear regression analysis and half maximal inhibitory concentration (IC_50_) of EF on COX activity in all three strains. See also Supplementary Table S2. (**c**) EF-mediated inhibition kinetics of COX in WT, MR, and SR strains using Lineweaver–Burk plots. (**d**) Depiction of proposed inhibitory targets of fumigants (EF and PH_3_), according to the degree of PH_3_ resistance in *S. oryzae* strains. The symbol ‘?’ indicates the unknown binding sites of the electron transfer chain (ETC) or other redox enzymes. In all experiments, protein samples were isolated from three independent replicates (*n* = 100 rice weevils) in each strain and each assay, and three biological replicates were performed for each experiment. All data represent mean ± standard deviation (SD).

To investigate the effect of PH_3_ fumigation on the respiratory efficiency of *S. oryzae* strains, we measured the activities of several enzymes as reference proteins in the PH_3_ susceptible WT and PH_3_–R strains (Fig. 1a). The cytochrome *c* oxidase (COX) activity of both PH_3_–R strains was higher than that of the WT strain, suggesting as positive correlation between COX activity and the degree of PH_3_ resistance. However, there was little difference in the activities of acetylcholinesterase (AChE) and carboxylesterase (CE) between the WT and PH_3_–R strains, although glutathione *S*-transferase (GST) activity was slightly lower in the SR strain than in MR and WT strains. In addition, these differential enzyme activity profiles demonstrated that the mode of action of PH_3_ in the PH_3_–R strains differs from that of organophosphates and carbamates, which affect the function of the nervous system of insects^22,23^.

To further investigate the extent of PH_3_ resistance in the WT and PH_3_–R strains, we performed the EF-mediated inhibition kinetics of these strains^24^. The inhibitory effect of EF as another fumigant on COX activity in each strain revealed that the half maximal inhibitory concentration (IC_50_) of the SR strain was not significantly different from that of the WT strain (Fig. 1b). In addition, the inhibition constant (K_i_) of the SR strain was lower than that of the WT strain under the same concentration of cytochrome used as the substrate (Supplementary Table S2). However, the MR strain did not show any significant change in IC_50_ and K_i_ values, compared with the WT strain. Intriguingly, the EF-mediated inhibition of COX activity in the WT strain was negligible, whereas that of PH_3_–R strains was pronounced in a concentration-dependent manner (Fig. 1c). Therefore, the discrepancy between the weak EF-mediated inhibition of COX activity and high Ct_99_ value of the disinfectant PH_3_ in the SR strain suggests that the acquisition of PH_3_ resistance in *S. oryzae* strains is not directly correlated with the degree of EF-mediated inhibition of COX activity *in vitro*, implying that resistance to PH_3_ in *S. oryzae* is partially gained through the perturbation of other redox proteins or metabolic sites in mitochondria (Fig. 1d).

### Distinct reorganization of cellular and mitochondrial metabolisms in PH_3_–R strains

**Figure 2:**
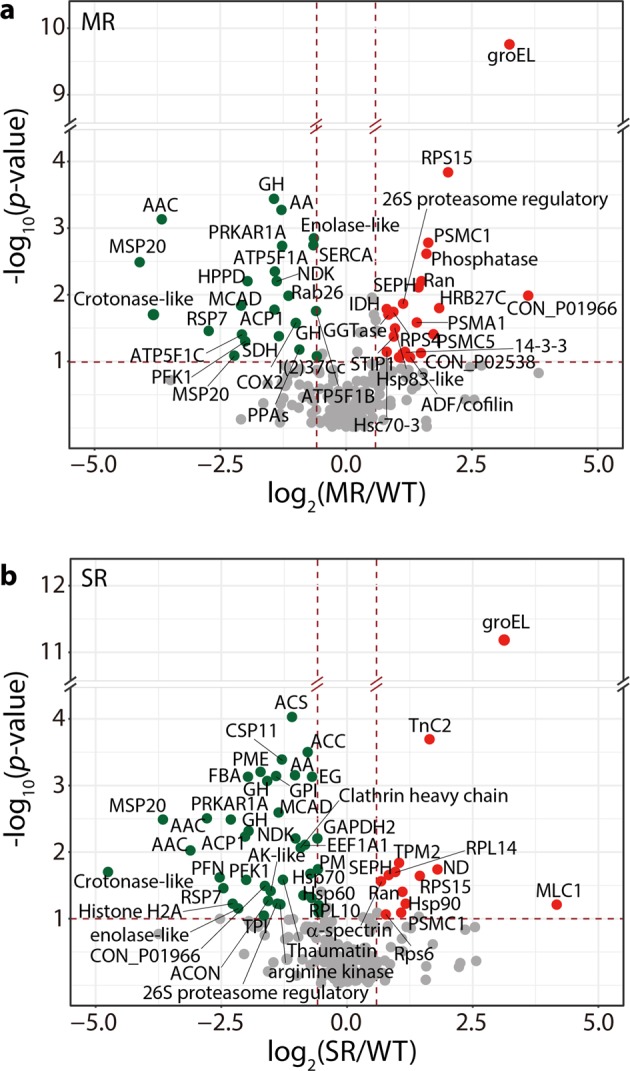
Volcano plots showing the fold-change and significance level of proteomes in the PH_3_–R strains. (**a**,**b**) Comparison of changes in gene expression (*Mann-Whitney* Test) as a function of protein accumulation in the WT (control) vs. MR strain (**a**) and WT vs. SR strains (**b**). The X-axis shows the fold-change, and the Y-axis shows the significance level. Red and green dots represent up-regulated and down-regulated genes, respectively. The horizontal brown dashed line marks the significance threshold (*p* < 0.1), and the vertical brown dashed line displays the value of 1.5-fold-change. Full description of the abbreviated protein names are listed in Supplementary Table S3.

To understand the metabolic consequences of PH_3_ exposure in both PH_3_–R strains of *S. oryzae*, we analyzed the difference in their proteome profiles using nLC-ESI-MS/MS (Fig. 2). Both PH_3_–R strains exhibited similar abundance patterns of several proteins such as the 40 S ribosomal protein, kinases, and glycoside hydrolase (Supplementary Table S3). Remarkably, however, the MR and SR strains showed differential abundance profiles of proteins affecting their mitochondrial metabolism and energy production including core metabolic enzymes involved in central metabolism, biosynthesis, cell signaling, and enzyme regulation (Fig. 2 and Supplementary Table S3). In the MR strain, 37 proteins showed differential abundance compared with the susceptible WT strain. Among these proteins, proteasome subunits, protease regulatory proteins, and stress responsive proteins (groEL, heat shock 70 kDa protein cognate 3, and stress-induced phosphoprotein 1) were highly abundant, whereas muscle specific proteins, ATP synthase subunits, and several mitochondrial proteins (including inorganic transport and ATP-ADP antiporter proteins) were less abundant in the MR strain than in the WT strain, indicating that an elevation in the stress response would be sufficient for the acquisition of moderate PH_3_ resistance (Fig. 2a and Supplementary Table S3). On the other hand, the SR strain exhibited a different protein abundance pattern, revealing that proteins involved in muscle contraction such as Ca^2+^-dependent troponin proteins and heat shock protein 90 (Hsp 90) were highly abundant (Fig. 2b and Supplementary Table S3). Moreover, the SR strain contained >2-fold higher levels of ND, a major component of the electron transport chain (ETC), troponin C, myosin light chain, and tropomyosin than the MR and WT strains. Remarkably, the mitochondrial ATP synthase and Ca^2+^-transporting ATPase were less abundant in the MR strain, whereas the level of metabolic enzymes involved in glycolysis and actin depolymerization was significantly lower in the SR strain than in the MR and WT strains (Fig. 2 and Supplementary Table S3). Overall, in *S. oryzae*, a substantial partitioning of the energy transduction system in the mitochondrial ETC and core metabolism occurs to induce PH_3_ resistance. In both PH_3_–R strains, the level of metabolic enzymes, involved in glycolysis and the oxidative tricarboxylic acid (TCA) cycle, was significantly suppressed (Fig. 2a). In particular, the level of ND was highly up-regulated in the SR strain (Fig. 2b).

**Figure 3:**
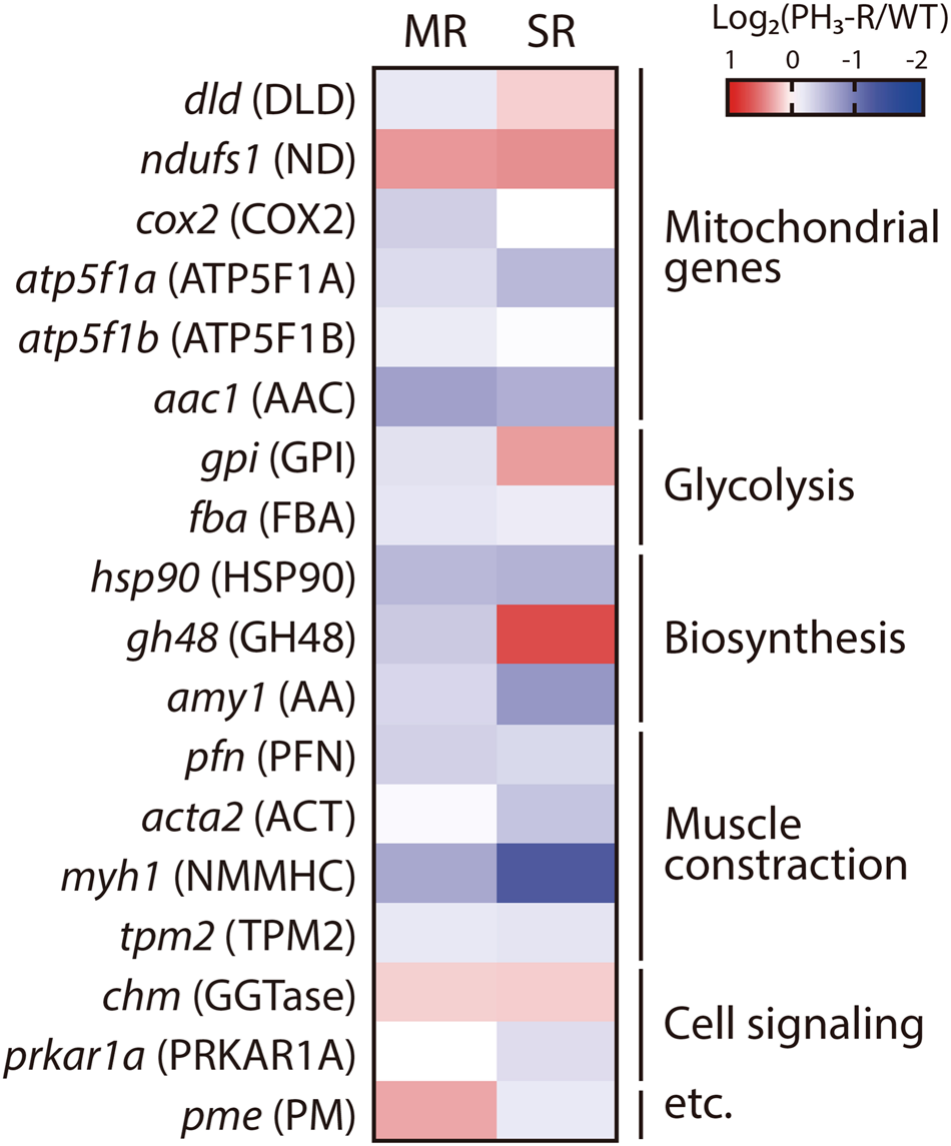
Differential expression patterns of a few selected genes in the WT, MR, and SR strains. Quantitative real-time (qRT)-PCR were performed in duplicate for every three independent biological replicates (n = 30). Gene expression levels were normalized by the expression of ribosomal protein L29 (*rpl29*) and were calculated using the 2^−∆∆Ct^ method. Heat map was constructed using Log2 gene expression ratio between the WT (control) and PH_3_–R strains. Each gene is represented by a gene name (protein name). *dld*, dihydrolipoamide dehydrogenase E3 subunit; *ndufs1*, NADH-ubiquinone oxidoreductase; *cox2*, cytochrome oxidase subunit II; *atp5f1a*, ATP synthase subunit alpha; *atp5f1b*, ATP synthase subunit beta; *aac1*, ADP, ATP carrier protein 1; *gpi*, glucose-6-phosphate isomerase; *fba*, fructose-bisphosphate aldolase; *hsp90*, heat shock protein 90; *gh48*, glycoside hydrolase family protein 48; *amy1*, alpha-amylase; *pfn*, Profilin; *acta2*, actin, muscle; *myh1*, myosin heavy chain 1; *tpm2*, tropomyosin-2; *chm*, Rab proteins geranylgeranyltransferase component A; *prkar1a*, cAMP-dependent protein kinase regulatory subunit; *pme*, pectin methylesterase; The primers used in this study are listed in Supplementary Table S4.

To further investigate whether either mitochondrial or core metabolism is correlated with PH_3_ resistance in *S. oryzae*, we analyzed the expression of 18 genes involved in PH_3_ resistance including several known reference genes by qRT-PCR (Fig. 3); these genes were selected because the nucleotide sequences of these genes or their homologs were available (Supplementary Table S4). The expression levels of the genes *ndufs1* and *chm* encoding ND and Rab protein geranylgeranyltransferase component A (GGTase), respectively, in both PH_3_–R strains were substantially higher than those in the WT strain. Indeed, both PH_3_–R strains showed relatively greater abundance of *chm* related to cellular signaling pathways regulating than the WT strain; this protein regulates normal cellular proliferation. By contrast, the expression level of the genes encoding ATP synthase subunit gamma (ATP5F1A), ADP, ATP carrier protein 1, Hsp90, alpha-amylase (AA), profilin (PFN), myosin heavy chain-1 (NMMHC), and fructose-bisphosphate aldolase (FBA) was down-regulated in both PH_3_–R strains (Fig. 3), implying that PH_3_ fumigation acts as a major selective pressure to change the core cellular metabolism that affects cellular energy production. The expression levels of *hsp90* and *amy1* involved in protein folding and carbohydrate metabolism, respectively, were down-regulated in both PH_3_–R strains. In addition, the expression levels of *acta2* and *myh1* in relation to muscle contraction were lower in the SR strain than in the MR and WT strains.

### Mitochondrial mutations in the SR strain

Our proteomic and qRT-PCR data suggest that both PH_3_–R strains share a common PH_3_ resistance mechanism to minimize the ATP-consuming metabolism by repressing core metabolism and modulating the magnitude of respiration. However, unlike the MR strain, the SR strain exhibited a distinct way of lowering substrate-level phosphorylation and mitochondrial respiration. To understand the metabolic discrepancy between the MR and SR strains with respect to cellular metabolism and bioenergetics, we sequenced the mtDNAs of the WT and PH_3_–R strains using next generation sequencing technology, and investigated whether the metabolic differences between the two PH_3_–R strains may be ascribed to the occurrence of SNP-like genetic mutations. A total of 15 point mutations were identified in nine genes including those encoding COX subunits 1 and 2 (*cox1* and *cox2*), ND subunits 1, 2, 4, 4 l, 5, and 6 (*nad1*, *nad2, nad4, nad4l, nad5*, and *nad6*), and ATP synthase subunit 6 (*atp6*) (Table 2). Marginal EF-mediated inhibition patterns of COX activity between the WT and PH_3_–R strains imply the occurrence of mutations in other genes encoding oxidative phosphorylation (OXPHOS) system (Fig. 1c). Although two SNPs were identified in the *cox1* and *cox2* genes in the SR strain, these mutations were silent (Table 2). Notably, a number of mtDNA point mutations (m.) were identified in the *nad4*, *nad5*, and *nad6* genes encoding protein subunits responsible for the assembly of ND in the respiratory chain: m.9082 C > A, m.8297 G > A, m.8231 G > A, m.8102 T > G in *nad4*; m.7551 C > T, m.7405 G > A, m.7353 C > G, m.6678 C > T in *nad5*, and m.10018 A > G in *nad6* (the base of the WT and MR strains > that of the SR strain). Among these, m.9082 C > A in *nad4*, m.7405 G > A and m.7353 C > G in *nad5*, and m.10018 A > G in *nad6* caused amino acid substitutions in the SR strain (Table 2 and Supplementary Fig. S1); serine (Ser; polar) to asparagine (Asn; polar), aspartic acid (Asp; acidic) to glutamic acid (Glu; acidic), and Asn (polar) to Ser (polar), respectively, while that in *nad4* caused a drastic change from alanine (Ala; non-polar) to the negatively charged Glu (Table 2 and Supplementary Table S5 and Fig. S1). In addition, the deduced amino acid sequences of the *nad* genes in *S. oryzae* were aligned to those of their counterparts in several model organisms (*Gallus gallus*, *Mus musculus*, *Homo sapiens*, and *Bos taurus*) and *Sitophilus zeamais* to identify conserved regions among different organisms, demonstrating that the m.9082 C > A in *nad4* is highly conserved with all identical sequence (Ala). The m.7405 G > A mutation in *nad5* showed similar sequences while other sites (m.7353 C > G in *nad5* and m.10018 A > G in *nad6*) are variable (Supplementary Fig. S2).

**Table 2:** Non-synonymous mutations in mitochondrial protein-coding genes of WT, MR, and SR strains of *Sitophilus oryzae*.

Gene name	Symbol	Strain	Strand	Nucleotide change^a^	Amino acid change^b^	Mutation type
Cytochrome c oxidase subunit 1	*cox1*	WT	Positive	1424 C	Ser	Silent
		MR		1424 C	Ser	
		SR		1424 T	Ser	
Cytochrome c oxidase subunit 2	*cox2*	WT	Positive	3201 C	Asp	Silent
		MR		3201 C	Asp	
		SR		3201 T	Asp	
NADH dehydrogenase subunit 1	*nad1*	WT	Negative	12330 G	Leu	Silent
		MR		12330 G	Leu	
		SR		12330 A	Leu	
NADH dehydrogenase subunit 2	*nad2*	WT	Positive	1108 A	Gly	Silent
		MR		1108 A	Gly	
		SR		1108 G	Gly	
NADH dehydrogenase subunit 4	*nad4*	WT	Negative	9082 C	Ala	Missense
		MR		9082 C	Ala	
		SR		9082 A	Glu	
		WT	Negative	8297 G	Met	Silent
		MR		8297 G	Met	
		SR		8297 A	Met	
		WT	Negative	8231 G	Leu	Silent
		MR		8231 G	Leu	
		SR		8231 A	Leu	
		WT	Negative	8102 T	Gly	Silent
		MR		8102 T	Gly	
		SR		8102 G	Gly	
NADH dehydrogenase subunit 4 L	*nad4l*	WT	Negative	9322 C	Leu	Silent
		MR		9322 C	Leu	
		SR		9322 T	Leu	
NADH dehydrogenase subunit 5	*nad5*	WT	Negative	7551 C	Leu	Silent
		MR		7551 C	Leu	
		SR		7551 T	Leu	
		WT	Negative	7405 G	Ser	Missense
		MR		7405 G	Ser	
		SR		7405 A	Asn	
		WT	Negative	7353 C	Asp	Missense
		MR		7353 C	Asp	
		SR		7353 G	Glu	
		WT	Negative	6678 C	Gly	Silent
		MR		6678 C	Gly	
		SR		6678 T	Gly	
NADH dehydrogenase subunit 6	*nad6*	WT	Positive	10018 A	Asn	Missense
		MR		10018 A	Asn	
		SR		10018 G	Ser	
ATP synthase subunit 6	*atp6*	WT	Positive	3949 T	Asn	Silent
		MR		3949 T	Asn	
		SR		3949 C	Asn	

^a^Nucleotide change, single nucleotide change compared with accession number NC_030765.1 in NCBI.

^b^Amino acid change, change in amino acid due to change in the codon sequence.

### Structural analysis of amino acid substitutions in the ND subunits

**Figure 4:**
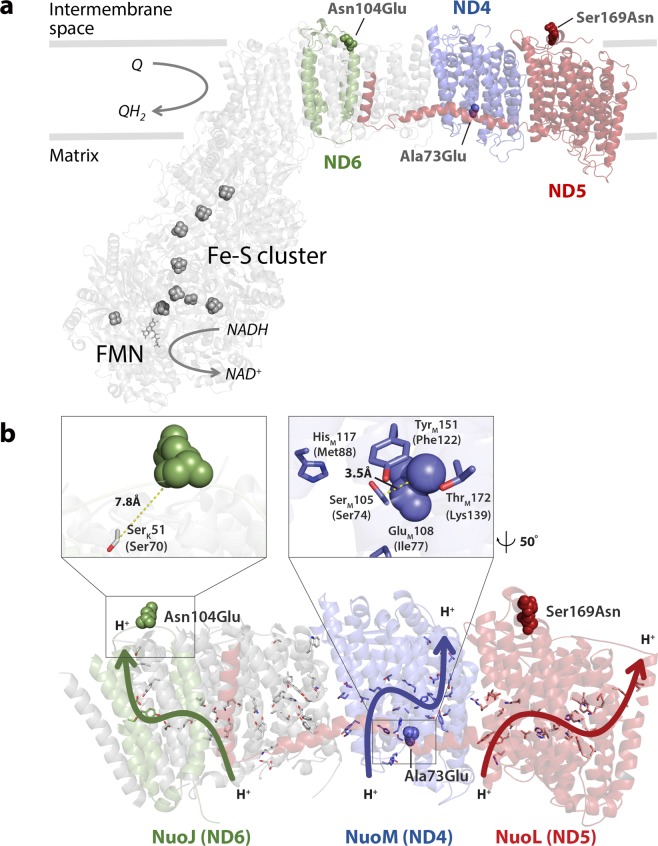
Schematic representation of the *S. oryzae* mitochondrial respiratory chain and putative mutation sites in mitochondrial (mt) genes associated with PH_3_ resistance in NADH dehydrogenase (ND). (**a**) Structure of ND (NADH–ubiquinone oxidoreductase) with three mt genes encoding ND4, ND5, and ND6 subunits (colored) modeled based on swine (PDB code 5GUP), bovine (5LC5), murine (6G2J), and bacterial (4HEA) complex I. Three mutation sites indicated by spheres are mapped onto their corresponding 3D model structures depicted as transparent ribbons. The iron-sulfur (Fe-S) cofactors of ND are depicted as gray spheres, and FMN cofactor as gray sticks. (**b**) Close-up views of the ND subunits (PDB 3RKO): nuoJ, nuoM, and nuoL (green, purple, and red, respectively) from *E. coli* BL21 (DE3). Polar residues along the channel are shown as sticks, with each arrow indicating the approximate proton translocation paths in *E. coli* ND. Distances from the mutation sites to adjacent polar residues are marked with yellow dotted lines.

To assess the potential impact of the identified missense mutations in *nad4*, *nad5*, and *nad6* genes on mitochondrial function, we constructed three dimensional (3D) models of the corresponding ND subunits, based on the protein structures of ND4 and ND5 subunits in *Mus musculus* (PDB No. 6G2J) and ND6 in *Thermus thermophilus* (PDB No. 4HEA) (Fig. 4a). We analyzed the predictive effects of mutations on the structure and function of respiratory chain complexes using the amino acid substitution algorithms (Table 3). These were combined to investigate the effect of mutations on the biological functions of proteins, with an improved prediction accuracy of >69%, when analyzed with SNPs associated with mitochondrial dysfunction^25^. Results using these three servers indicated that the amino acid substitution in ND4 was deleterious, while that in ND6 was relatively neutral. Subsequently, three substitutions (Ala73Glu in ND4, Ser169Asn in ND5, and Asn104Glu in ND6) identified in the SR strain were mapped onto their model structures (Fig. 4a,b). The membrane-bound domain of ND is involved in proton translocation across the membrane^26^. All three mutation sites in the model structures of *S. oryzae* ND were superimposed with polar amino acid residues, potentially involved in proton translocation chains of *Escherichia coli* NuoJ, NuoM, and NuoL subunits with the root-mean-square-deviation values of 4.875 Å, 1.367 Å, and 1.191 Å, respectively (Fig. 4b). Remarkably, the Ala73Glu substitution in ND4 was only 3.5 Å from a residue in *E. coli* ND acting as a proton entrance, whereas the Asn104Glu substitution in ND6 was 7.8 Å away from the proton exit of ND (Fig. 4b).

**Table 3:** Structural classification and pathogenic prediction of mutations in mitochondrial protein-coding genes of *S. oryzae*.

Protein subunit	Amino acid substitution (nucleotide mutation)	Predictions					
		SIFT		PROVEAN		Polyphen-2	
		Score^a^	Prediction	Score^b^	Prediction (cutoff = -2.5)	Score^c^	Prediction
ND4	A73E (m.9082 C > A)	0.00	Damaging	−4.070	Deleterious	0.998 (0.27;0.99)^d^	Probably damaging
ND5	S169N (m.7405 G > A)	0.36	Tolerated	−0.889	Neutral	0.395 (0.90;0.90)	Benign
ND6	N104E (m.10018 A > G)	0.27	Tolerated	−2.033	Neutral	0.026 (0.95;0.81)	Benign

^a^SIFT score ranges from 0 to 1. Amino acid substitutions with a SIFT score ≤ 0.05 are predicted as “damaging”, and those with SIFT score > 0.05 are predicted as “tolerated”.

^b^Variants with a PROVEAN score ≤ −2.5 are considered “deleterious”, and those with a PROVEAN score > −2.5 are considered “neutral”.

^c^Conservation of a position in the multiple sequence alignment, and deleterious effect on the protein structure results in the Position-Specific Independent Count (PSIC) score ranging from 0 to 1. Non-synonymous SNPs are classified as “possibly damaging” or “probably damaging” (PSIC >0.5) or “benign” (PSIC < 0.5)

^d^(sensitivity; specificity).

## Discussion

The response of redox-active DLD, a core metabolic enzyme, to PH_3_ differed between the PH_3_–R strains and WT strain in such a way that the P49S mutation in the *rph*2 locus contributes to PH_3_ resistance in association with a synergistic *rph*1 locus^18^. This coincides with the observation that the P49S substitution in DLD is frequently found in PH_3_–R strains of *R. dominica* and *T. castaneum* from India and Australia in the absence of PH_3_ fumigation^10^, indicating that PH_3_ fumigation acts as a selection pressure that generates PH_3_–R insect pests by altering the activity of core metabolic enzymes. This is further supported by the recent finding that *rph*l variants identified in the PH_3_–R strains of *R. dominica*, *S. oryzae*, *C. ferrugineus*, and *T. castaneum* share common mutations in an orthologous gene encoding a cytochrome *b*5 fatty acid desaturase (Cyt-b5-r)^13^. Mutations in Cyt-b5-r in PH_3_–R insects limit the potential for lipid peroxidation through reactive oxygen species generated by DLD. Moreover, a proteomic study revealed that a PH_3_–R strain of *R. dominica* exhibited PH_3_ resistance by altering the expression of 21 proteins involved in the TCA cycle and glycolysis^21^.

In this study, we found two distinct PH_3_–R *S. oryzae* strains (MR and SR) that employed different strategies to develop PH_3_ resistance (Table 1 and Fig. 1). The potential target of PH_3_ seems to be the energy transduction system including the ETC located in the mitochondrial membranes of eukaryotic cells^27^. The PH_3_-mediated inhibition of COX activity interferes with energy production, rendering insects incapable of performing various functions because of the shortage of ATP^28,29^. In this study, both PH_3_–R strains showed slightly higher COX activity than the susceptible WT strain. However, the IC_50_ values of EF in these strains was not proportional to the extent of PH_3_ resistance (Fig. 1a,b), which is consistent with EF-mediated inhibition of COX activity^30^. However, the different magnitude of EF-mediation inhibition of COX activity in *S. oryzae* strains (i.e., WT and both R strains) may be caused by additional mtDNA mutations as well as changes in expression of genes encoding other energy transducing proteins (Fig. 1c and Table 2). Our data suggest that moderate PH_3_ resistance may be acquired by the modulation of expression levels of core metabolic enzymes, without mutations in the *dld* gene (Fig. 1a and Table 1 and Supplementary Table S1). The SR strain in the DLD mutation background exhibited extraordinary PH_3_ resistance when compared with the MR and WT strains (Table 1 and Supplementary Table S1). Overall, the energy transduction system in mitochondria and core metabolism seem to undergo a substantial metabolic partitioning, suggesting that reprogramming activities involved in metabolism and respiratory chains, such as altered bioenergetics, suppressed biosynthesis, and redox balance improves cellular fitness and provides a selective advantage during PH_3_ fumigation. Recent studies on *C. ferrugineus* and *R. dominica* suggest that specific core metabolisms and the mitochondrial ETC are highly associated with PH_3_ resistance^3,5,6^. Although the general PH_3_ resistance mechanisms in insects are related to target site insensitivity, increases in detoxifying enzyme levels, behavioral modifications, and physiological alterations (Fig. 1)^31–34^, the basis of the induction of PH_3_ resistance in insects, and the molecular and genetic bases of energy modulation by insect pests under life-threatening conditions remain unclear.

**Figure 5:**
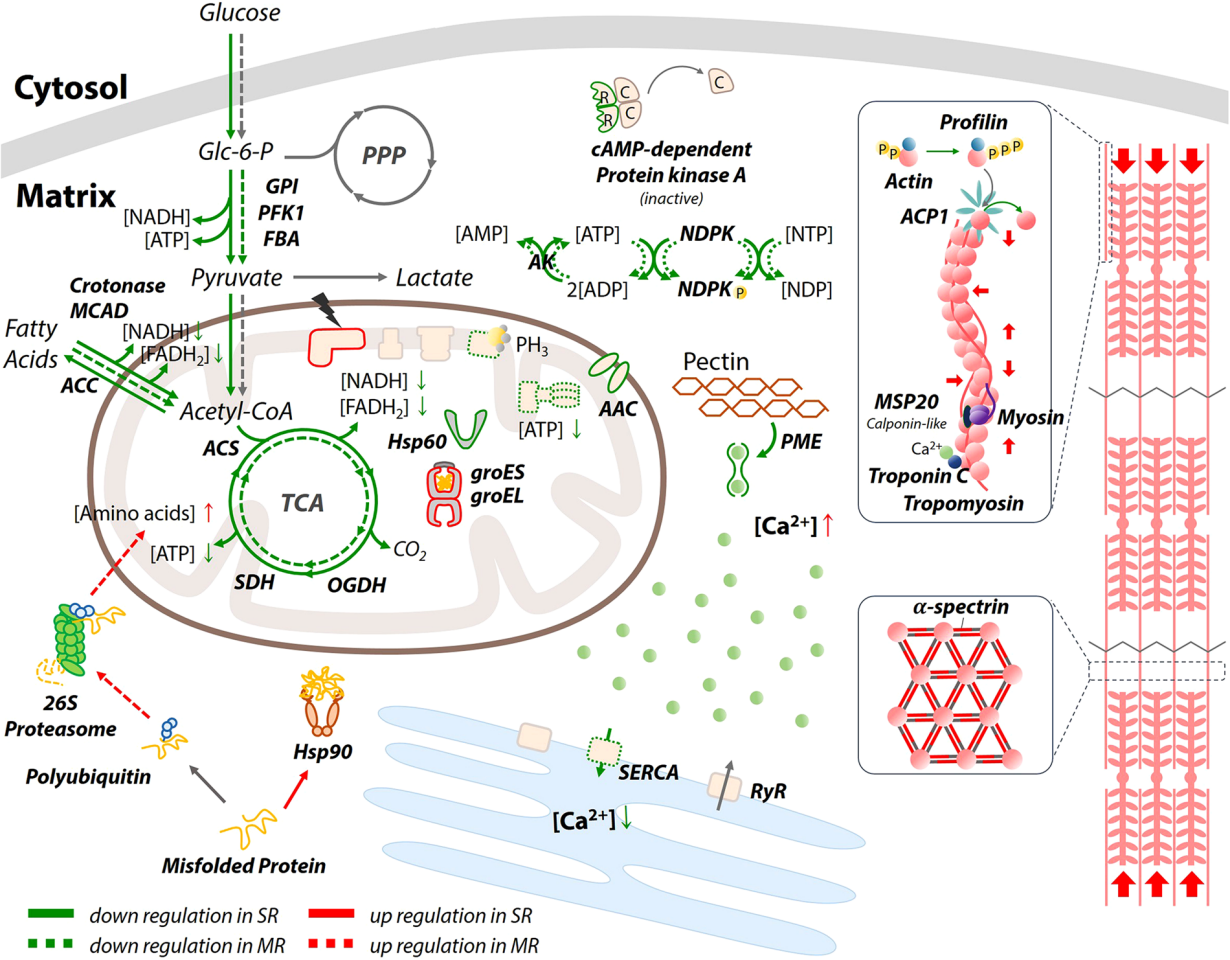
Reprogrammed metabolic pathways in the PH_3_–R strains of *S. oryzae*. GPI, glucose-6-phosphate isomerase; PFK1, 6-phosphofructokinase; FBA, fructose-bisphosphate aldolase; MCAD, putative medium-chain specific acyl-CoA dehydrogenase; ACC, acetyl-CoA carboxylase; ACS, ATP-citrate synthase; SDH, succinate dehydrogenase; OGDH, 2-oxoglutarate dehydrogenase E1; Hsp 60 and 90, heat shock protein 60 and 90; groES, chaperonin groES; groEL chaperonin groEL; AAC, ADP/ATP carrier protein; SERCA, the sarcoplasmic/endoplasmic reticulum Ca^2+^-ATPase; RyR, ryanodine receptor; AK, adenylate kinase; NDPK, nucleoside-diphosphate kinase; PME, pectin methylesterase; ACP1, actin-interacting protein; MSP20, Muscle-specific protein 20.

Proteome profiles of PH_3_–R strains indicate that reprogrammed core metabolism and mitochondrial function including the respiratory chains are the basis of PH_3_ resistance (Fig. 2). The MR and SR strains exhibited a reduction in glycolytic flux as well as in the level of glycolytic intermediates to suppress subsidiary pathways, resulting in reduced metabolic demands (Fig. 5). In addition, the level of TCA cycle intermediates, which serve as precursors for macromolecule biosynthesis, was also reduced, which is consistent with a classical example of a reprogrammed metabolic pathway in cancer cells such as the Warburg effect or aerobic glycolysis^35^. Intriguingly, the SR strain shares several common metabolic features with cancer cells. The primary characteristic of cancer cell is the avoidance of OXPHOS. Instead, cancer cells utilize substrate-level phosphorylation through aerobic glycolysis and lactate production, regardless of oxygen availability, resulting in acidosis within cells^36,37^. Similarly, ND was impaired in the SR strain because of missense mutations in genes encoding ND subunits 4–6 (Table 3 and Fig. 4), presumably resulting in the modulation of energy production during aerobic glycolysis as well as mitochondrial metabolism for lactate fermentation with substrate-level phosphorylation. This indicates that the inhibition by PH_3_ exposure could be compensated by activating other proteins for alternative respiration, which might be more favorable for cellular survival under PH_3_ exposure (Figs 2b and 5). Despite the incredible genetic and histological heterogeneity of PH_3_–R strains, resistance to PH_3_ might be ascribed to the common suppression of a finite set of pathways to support core functions such as anabolism, catabolism, and redox balance (Figs 2 and 5)^38^. The general repression of these pathways may reflect their regulation by signaling pathways and cellular metabolism, which were perturbed in both PH_3_–R strains (Fig. 5). This conjecture is further supported by the proteome profiles of PH_3_–R strains, which indicated that highly abundant proteins such as troponin, Hsp90, and mutated ND4 in the PH_3_–R strains of *S. oryzae* are closely associated with the capacity to transition between aerobic and anaerobic respiration, in relation to the modification of OXPHOS (Fig. 2 and Tables 2 and 3). An anaplerotic flux in muscle tissues appears to be activated in the MR strain, whereas the SR strain clearly exhibited a distinct means to induce Ca^2+^-mediated muscle contraction by actively pumping Ca^2+^ back into the sarcoplasmic reticulum (Fig. 5). It is known that troponin C plays a key role in cardiac muscle contraction^39^ by controlling Ca^2+^-mediated regulation of interactions between actin and myosin. In this regard, our proteomic data suggest that the SR strain possesses a mechanism for muscle contraction regulated by troponin C, which is a highly selective marker for myocardial infarction and heart muscle cell death in human^40^. Taken together, the MR strain of *S. oryzae* minimized the metabolic burden by bypassing ATP consuming pathway, whereas the SR strain reorganized the energy transduction system to seemingly undertake anaerobic respiration, regardless of the active COX, during PH_3_ fumigation. This change in energy production confers a respiratory advantage against PH_3_ fumigation. It is likely that mutations in ND subunits abolish OXPHOS to avoid the acute toxicity of PH_3_. Thus, if impaired mitochondrial energy transducing activities benefit the PH_3_ resistance of *S. oryzae*, some of them may be suitable as therapeutic targets. Indeed, mitochondrial mutations affecting OXPHOS efficacy confers drug resistance in malaria parasites^41,42^.

This plausible PH_3_ resistance mechanism was further reinforced by mtDNA sequencing. In the SR strain, several missense mutations in *nad4, nad5*, and *nad6* genes encoding ND subunits and silent mutations in *cox1* and *cox2* genes were identified (Table 2). The structural analysis of these mutation sites suggests that Ala73 in ND4 of *S. oryzae* plays an important role in proton uptake, indicating that the Ala73Glu substitution is responsible for altering the proton translocation efficiency (Fig. 4b). Therefore, the m.9082 C > A mutation has a drastic effect on structure and function of the mitochondrial ND, suggesting that the mitochondrial ND of the SR strain is strongly associated with PH_3_ resistance through muscle contraction by Ca^2+^ pumping in response to PH_3_ fumigation. Moreover, proteins involved in stress response, biosynthesis, transport, and signaling were also differentially expressed in the MR and SR strains (Fig. 2), implying that PH_3_ fumigation functions as a major selection pressure to change the cellular metabolism in rice weevil. This is also supported by the proteomic results of COX2 expression, which was approximately 2-fold lower in the MR strain than in the susceptible WT strain, which is consistent with the qRT-PCR data (Fig. 3 and Supplementary Table S3). However, these data suggest that the inhibition of COX activity observed in the PH_3_–R strains is not well correlated with the acquisition of PH_3_ resistance (Fig. 1c,d), implying either the presence of various COX isozymes or an impaired OXPHOS system in PH_3_–R strains caused by mtDNA mutations. Furthermore, the high abundance of Hsp90 and groEL/ES, another characteristic marker of cancer cells, likely explains the development of PH_3_ resistance in *S. oryzae* strains (Fig. 2 and Supplementary Table S3). To our knowledge, this is the first report of mutations in ND subunits of insect pests that can be a resistant factor to the redox-active gas, phosphine. Therefore, to effectively manage PH_3_–R insect pests, fumigants that target proteins other than those involved in mitochondrial energy production should be considered.

## Conclusions

Both PH_3_–R strains exhibited higher resistance to EF-mediated inhibition of COX than the WT, whereas the disinfectant Ct of the SR strain was much longer than that of the MR strain. Unlike the MR strain, which primarily showed changes in the expression levels of genes encoding metabolic enzymes involved in catabolic pathways that minimize metabolic burden, the SR strain showed changes in the mitochondrial respiratory chain. We found that the acquisition of strong PH_3_ resistance necessitates the avoidance of OXPHOS via the introduction of a few non-synonymous mutations in mitochondrial genes encoding ND as well as nuclear genes encoding DLD, concomitant with metabolic reprogramming, a recognized hallmark of cancer metabolism. These results suggest that anaerobic respiration is the survival strategy that SR insect pests use to compensate for minimized energy transduction under anoxic conditions. Taken together, PH_3_ toxicity acts as a selection pressure that not only alters cellular metabolism but also modulates energy transduction via mitochondrial mutations; this explains the mechanism of PH_3_ resistance in *S. oryzae*.

## Methods

### Insect strains and growth conditions

The susceptible WT strain of *S. oryzae* was obtained from Murdoch University (Perth, Australia) and maintained under pesticide-free conditions. The MR strain was obtained from the central grain storage (JaeHee RPC, Gunsan, Korea) in 2016. The SR strain was collected from Chungbuk National University (Cheongju, Korea) in 2015. All stock colonies of *S. oryzae* were successively cultured on rice grains at the Plant Quarantine Technology Center in Korea under controlled conditions (25 ± 1 °C temperature, 80% relative humidity, and 16 h light/8 h dark photoperiod).

### Fumigation assay

Adults of *S. oryzae* were placed on brown rice in a plastic dish (Φ 10 cm × 4 cm; SPL Life Science, Pocheon, Korea) with a center-opened cap covered by nylon net. The toxicity of PH_3_ against *S. oryzae* was tested using a series of concentrations from 0.01 to 1.0 mg/L in 12 L desiccators (Bibby Scientific, Staffordshire, UK) sealed with glass stoppers for 20 h at 20 °C. PH_3_ (ECO_2_Fume™; 2% PH_3_ + 98% CO_2_) was obtained from Cytec (Sydney, Australia). All experiments were performed with 30 insects in triplicate. The desiccator was furnished with a lid fitted with a septum injection system (Alltech Crop Science, Nicholasville, KY). Each desiccator was measured for its volume prior to fumigation bioassay by weighing the amount of water at 20 °C. A magnetic bar placed at the bottom of the desiccator was used to properly stir the gas for even distribution. To determine residual concentrations of PH_3_ in the desiccator, a gas was sampled at 10 min, 1 h, 3 h, 6 h, and 20 h post PH_3_ fumigation and stored in a gas sampling bag (1-L Tedlar®, SKC, Dorset, United Kingdom). Gas chromatography (GC) analysis performed using an Agilent GC 7890 A coupled with a flame photometric detector (FPD) and a HP-PLOT/Q column (30 m length × 530 µm internal diameter × 40 µm film) (Agilent, Santa Clara, CA). Detailed condition of GC analysis can be found in Supplementary Methods.

### Determination of the Ct value for PH_3_

The concentrations of PH_3_ measured during the exposure periods were used to determine the Ct values, as described in^43^. Detailed equation of Ct values can be found in Supplementary Methods.

### Measurement of enzyme activities

Protein samples were isolated from three independent replicates (*n* = 100) and were performed according to the method described in Supplementary Methods. Activities of COX, AchE, CE, and GST were determined using the methods reported previously by Nathanailides and Tyler^44^, Ellman *et al*.^45^, Mackness *et al*.^46^, and Habig and Jakoby^47^, respectively. Each assay was performed in triplicate. Enzyme activities were expressed as units/mg protein. Data were expressed as mean ± standard deviation. The results were analyzed by one-way analysis of variance (ANOVA) and Tukey’s post-hoc test using SPSS statistics version 23.0.

### EF inhibition kinetics of COX

To study the inhibitory effect of EF on COX in different PH_3_–R strains of *S. oryzae*, the mitochondrial fraction of each insect was exposed to 0, 1, 5, 10, 50, and 75 mM EF. The activity of COX was measured as described above. The IC_50_ value and the inhibition constant (K_i_) of EF was calculated by least-squares fit dose-response curves and enzyme kinetics-inhibition modes using GraphPad Prism version 8.0.1 for Windows (La Jolla, CA). Reduced cytochrome *c* was used as a substrate for COX in each strain at different concentration (0.011, 0.015, 0.022, 0.044 and 0.055 mM) in the presence of 0, 1, and 10 mM EF.

### Proteomic analysis using nLC-ESI-MS/MS

Proteomic analysis was performed using a Thermo Scientific Q Exactive Hybrid Quadrupole-Orbitrap instrument (Thermo Fisher Scientific Inc., Waltham, MA) with a Dionex U 3000 RSLC nano high performance liquid chromatography (HPLC) system. An ESI source fitted with a fused silica emitter tip (New Objective, Woburn, MA) was employed with a mobile phase consisting of the water:acetonitrile (98:2 [v/v]) containing 0.1% formic acid. The trypsin-treated samples were trapped on an Acclaim PepMap 100 trap column (100 μm × 2 cm, nanoViper C18, 5 μm, 100 Å) and washed for 6 min at a flow rate of 4 μL/min, and then separated on an Acclaim PepMap 100 capillary column (75 μm × 15 cm, nanoViper C18, 3 μm, 100 Å) at a flow rate of 300 μL/min. The resulting peptides were electrosprayed through a coated silica tip with ion spray voltage of 2,000 eV. Mass data were collected and analyzed using Proteome Discoverer 1.4, MaxQuant 1.6, and Scaffold 4.8.4 against the protein databases of *S. oryzae* and *T. castaneum*. Detailed information including analysis conditions and data processing can be found in Supplementary Methods.

The expression of 18 genes expected to be associated with PH_3_ resistance from proteome analysis were validated by qRT-PCR. The method of qRT-PCR was in Supplementary Methods.

### mtDNA sequencing and annotation

Total DNA, including mtDNA, was extracted from 30 individuals of each strain using the QIAamp DNA Mini Kit (Qiagen, Dusseldorf, Germany). Short-read assembly was performed using SOAPdenovo^48^, and scaffolding was performed with a minimum size of 100 bp. The assembled scaffolds and contigs (≥100 bp) were mapped to the sequences of *Sitophilus oryzae* using the National Center for Biotechnology Information (NCBI) BLASTN tool with default parameters. Contigs and scaffolds with query coverage greater than 40% were retrieved and used to search the non-redundant nucleotide and protein databases using BLASTN (http://blast.ncbi.nlm.nih.gov/). All raw reads were realigned with the assembled sequences using the Burrows-Wheeler Aligner (BWA) software. The aligned paired-end reads were used to determine the sequencing depth. A second round of assembly was carried out using the initially assembled contigs and scaffolds. Contigs and scaffolds with an overlap of ≥12 bp were assembled into larger scaffolds based on synteny between the assembled and reference genomes, we further joined them into larger scaffolds.

The mtDNA sequence was annotated using the MITOS web server^49^. Nucleotide sequences of protein-coding genes were translated into amino acid sequences using the genetic code for invertebrate mitogenomes. The predictions from MITOS were manually curated using other published skipper mitogenomes as references, and the starts and ends of genes were modified, if necessary, to be consistent with other species. The new open reading frames of the protein-coding genes (after modification) were validated.

### Structural mapping and analysis

Three non-synonymous mutations in mt genes encoding the ND subunits were mapped onto their corresponding rice weevil and bacterial model structures. Each subunit model, comprising *S. oryzae* ND, was constructed using the SWISS-MODEL server with the available high-resolution ND subunit structures of the swine (PDB accession code 5GUP for ND1 and ND3), bovine (5LC5 for ND2 and ND4L), murine (6G2J for ND4 and ND5), and bacterial (4HEA for ND6) complex I as template structures.

To determine the effect gene mutations on the structure and function of ND subunits, amino acid substitutions (AAS) analysis was performed using predictive approaches such as SIFT (http://sift.jcvi.org), PROVEAN (http://provean.jcvi.org), and Polyphen-2 (http://genetics.bwh.harvard.edu/pph/) web servers.

## Supplementary information

Supplementary information

## Data Availability

The data that support the findings of this research work are available from the corresponding author upon request.
