# Supplementary material for: Minimization of energy transduction confers resistance to phosphine in the rice weevil, Sitophilus oryzae
Source: Sci Rep. 2019 Oct 10;9:14605. doi: 10.1038/s41598-019-50972-w (PMC6787191; doi:10.1038/s41598-019-50972-w)
Supplement: Supplementary file 1 — Supplementary information [file 41598_2019_50972_MOESM1_ESM.docx]

**Supplementary Information**

**Minimization of energy transduction confers resistance to phosphine in the rice weevil, *Sitophilus oryzae***

Kyeongnam Kim^1,§^, Jeong Oh Yang^2, §^, Jae-Yoon Sung^3^, Ji-Young Lee^3^, Jeong Sun Park^2^, Heung-Sik Lee ^2^, Byung-Ho Lee^4^, Yonglin Ren^5^, Dong-Woo Lee^3,*^, and Sung-Eun Lee^1,*^

^1^School of Applied Biosciences, Kyungpook National University, Daegu 41566, Korea.

^2^Animal and Plant Quarantine Agency (APQA), Gimcheon 39660

^3^Department of Biotechnology, Yonsei University, Seoul 03722, Korea.

^4^Institute of Agriculture and Life Science, Gyeongsang National University, Jinju 52828, Korea.

^5^School of Veterinary and Life Science, Murdoch University, 90 South St. Murdoch, WA 6150, Australia.

*Corresponding authors

**Sung-Eun Lee,** School of Applied Biosciences, Kyungpook National University, Daegu 41566, Korea.

Tel.: +82-53-950-7768; Fax: +82-53-953-7233; E-mail: [selpest@knu.ac.kr](mailto:selpest@knu.ac.kr).

**Dong-Woo Lee,** Department of Biotechnology, Yonsei University, Seoul 03722, Korea

Tel.: +82-2-2123-2886; Fax: +82-2-362-7265; E-mail: [leehicam@yonsei.ac.kr](mailto:leehicam@yonsei.ac.kr)

^§^These authors contributed equally to this work.

**Table of Contents**

1. **Supplementary Methods**

Chemicals

Sequencing of the *dld* gene

Measurement of PH_3_ concentration

Determination of the concentration-time (Ct) value for PH_3_

Protein extraction

Protein preparation and proteomic analysis using nLC-ESI-MS/MS

Data processing

Quantitative real-time (qRT-PCR)

1. **Supplementary Tables**

**Table S1.** Alignment of dihydrolipoamide dehydrogenase (DLD) of PH_3_ susceptible (WT) and resistant (R1 and R2) strains

**Table S2.** Kinetic parameters of COX in WT, MR, and SR strains upon exposure to ethyl formate.

**Table S3.** Fold-change in protein levels in WT and PH_3_-R strains using nLC-ESI-MS/MS.

**Table S4.** List of qRT-PCR primers used in this study

**Table S5.** Mitochondrial genetic code of *S. oryzae*.

1. **Supplementary Figures**

**Figure S1.** Mitochondrial gene sequences carrying point mutations in the S, MR, and SR strains.

**Figure S2.** Alignments of deduced amino acid sequences of mtDNA sequences encoding ND subunits with point mutations.

1. **References**

**1. Supplementary Methods**

**Chemicals**

Phosphine (PH_3_; ECO_2_Fume™; 2% PH_3_ + 98% CO_2_) was obtained from Cytec (Sydney, Australia). Acetylthiocholine iodide (ATChI), bovine serum albumin (BSA), 1-chloro-2,4-dinitrobenzene (CDNB), cytochrome c, 5,5′-dithiobis(2-nitrobezoic acid), ethyl formate (97% purity), Fast Blue B salt, reduced L-glutathione, and 1-naphthyl acetate (α-NA) were purchased from Sigma-Aldrich (St. Louis, MO). DEPC-treated water was purchased from Biosesang (Seongnam, Korea). The DNeasy blood and tissue kit and QIAamp DNA Mini Kit were purchased from Qiagen (Valencia, CA), and the Maxima First Strand cDNA Synthesis Kit with dsDNase was purchased from Thermo Fisher Scientific (Waltham, MA). TRIzol^®^ Reagent was purchased from Ambion (Austin, TX) and Luna^®^ Universal qPCR Master Mix was purchased from New England Biolabs (Ipswich, MA).

**Sequencing of the *dld* gene**

Total DNA was extracted from the two hind legs of *S. oryzae* insects randomly selected from each strain. The DNeasy Blood and Tissue Kit (Qiagen, Valencia, CA) was used for DNA isolation, according to the manufacturer’s instructions. The *dld* gene was amplified by PCR using primers adapted from [1]: So_dld_F (5′-AGGAGTACGGCGCATCA-3′) and So_dld_R (5′-CGATAACAAAAAAGGGGCG-3′). PCR was performed using AccuPower PreMix (Bioneer, Daejeon, Korea) under the following conditions: initial denaturation at 94°C for 5 min, followed by 30 cycles at 94°C, 52°C, and 72°C for 1 min each, and a final extension at 72°C for 7 min. DNA sequencing was conducted using ABI PRISM® BigDye® Terminator v3.1 Cycle Sequencing Kit with an ABI 3100 Genetic Analyzer (Applied biosystems, Foster City, CA). All PCR products were sequenced in both directions. Sequences of both strands from each strain were aligned using the Clustal Omega program (https://www.ebi.ac.uk/tools/msa/clustalo) to obtain a consensus sequence for each strain. Sequences were translated using the EMBOSS Transeq (https://www.ebi.ac.uk/tools/st/emboss_transeq).

**Measurement of PH_3_ concentration**

To determine residual concentrations of PH_3_ in the desiccator, a gas was sampled at 10 min, 1 h, 3 h, 6 h, and 20 h post PH_3_ fumigation and stored in a gas sampling bag (1-L Tedlar®, SKC, Dorset, United Kingdom). Gas chromatography (GC) analysis was performed using an Agilent GC 7890A coupled with a flame photometric detector (FPD) and a HP-PLOT/Q column (30 m length × 530 µm internal diameter × 40 µm film) (Agilent, Santa Clara, CA). This equipment was operated in split mode (10:1). The temperature of the injector and oven was set at 200°C and that of the detector at 250°C. The injection volume was 20 µL and the flow rate was 5 mL/min. The residual concentration of the fumigant was calculated according to the obtained peak areas vs. external standards.

**Determination of the concentration-time (Ct) value for PH_3_**

Concentrations of PH_3_ were monitored during the exposure periods and used to determine the concentration-time (Ct) values according to Eq. 1 [2] as follows:

Ct = ∑(C_i_+C_i+1_) (t_i+1_- t_i_)/2 Eq. 1.

where: C is the PH_3_ concentration (mg/L)

t is the time of exposure (h); and i is the order of measurement.

Therefore, Ct value is based on concentration × time (mg h/L).

The toxicity of PH_3_ against *S. oryzae* was monitored at least three different Ct values using Excel v. 2010, and the concentration × time values for 50% mortality (Ct_50_) and 90% mortality (Ct_90_) were calculated. The time values for 50% and 90% mortality rates were determined using Probit analysis (SPSS version 23.0).

**Protein extraction**

Protein samples were isolated from three independent replicates. Individuals of each strain were collected using a standard testing sieve (10 mesh) to remove rice and frozen immediately at −70°C. The frozen insects (0.01 g; approximately 100 individuals) were ground in liquid nitrogen using a mortar and pestle. Each ground sample was then transferred to a Dounce glass homogenizer, followed by the addition of Tris-buffer (pH 7.4) containing 500 mM sucrose, and homogenized again. The homogenized solution was filtered through a 0.4-μm cell strainer (SPL Life Science, Pocheon, Korea) to remove the chitinous skin and cell debris. The filtrates were centrifuged at 600 × *g* at 4°C for 10 min. The supernatant (crude protein extract) was collected and re-centrifuged at 10,000 × *g* at 4°C for 15 min. The resulting pellet containing mitochondria was named the mitochondrial fraction, and the supernatant containing the soluble cytosol was designated as the S9 fraction. Protein was quantitatively measured using the Protein Assay Dye Reagent Concentrate (Bio-Rad, Hercules, CA), and the protein standard curves were constructed using different concentrations of BSA, according to the manufacturer’s instructions.

**Protein preparation and proteomic analysis using nano-LC-ESI-MS/MS**

The crude protein extract containing the mitochondrial fraction was obtained from 50 rice weevils, following the same procedure mentioned in section 2.4. The Tris-buffer with 500 mM sucrose (pH 7.4) and protease inhibitor cocktail Set I (Calbiochem, San Diego, CA) was used to avoid protein degradation during protein extraction. The protein extracts were denatured by incubating them with 50 mM ammonium bicarbonate buffer (pH 7.8) and 6 M Urea for 3 h at room temperature. The denatured protein extracts were incubated with 10 mM dithiothreitol for 2 h at room temperature in order to reduce their disulfide bonds. Finally, the reduced protein extracts were placed to react with iodoacetamide (IAA) for 1 h, followed by trypsin treatment at 37℃ in a shacking incubator for 18 h. The treated samples were desalinated using the Sep-Pac C18 cartridges (Waters co., Milford, MA) according to the manufacturer’s procedures.

The proteomic analysis was used a Thermo Scientific Q Exactive Hybrid Quadrupole-Orbitrap instrument (Thermo Fisher Scientific Inc., Waltham, MA) with a Dionex U 3000 RSLC nano-HPLC system. An electrospray ionization source (ESI) with a fused silica emitter tip (New Objective, Woburn, MA) was employed with a mobile phase consisting of the water/acetonitrile (98:2 v/v) solution containing 0.1% formic acid. The trypsin-treated samples were trapped on an Acclaim PepMap 100 trap column (100 μm × 2 cm, nanoViper C18, 5 μm, 100 Å) and washed for 6 min at a flow rate of 4 μL/min, and then separated on an Acclaim PepMap 100 capillary column (75 μm × 15 cm, nanoViper C18, 3 μm, 100 Å) at a flow rate of 300 μL/min. The resulting peptides were electro-sprayed through a coated silica tip with ion spray voltage of 2,000 eV. The mass data were collected and analyzed using Proteome Discoverer 1.4, MaxQuant 1.6, and Scaffold 4.8.4 against the protein databases of *S. oryzae* and *T. castaneum*.

**Data processing**

Results of MaxQuant 1.6 were imported into Scaffold Q+ v4.6.1 (Proteome Software) using intensity-based analysis (centroided peak intensity) with peptide thresholds of 1.0% false discovery rate (FDR), protein thresholds of 1.0% FDR, and at least 2 peptides per protein. Analysis was performed both with and without the inclusion of non-exclusive peptides where unique peptides are defined as those associated with a single protein group. Results were expressed as log2 ratios and downloaded as Excel files. Statistical comparisons of each pair of isolates were performed within Scaffold using the Mann-Whitney test with Benjamini-Hochberg correction for multiple testing; the software also provided log2 ratio for each pairwise comparison ([volcano plot](https://journals.plos.org/plosone/article?id=10.1371/journal.pone.0190836#pone.0190836.s001)).

**Quantitative real-time PCR (qRT-PCR)**

Thirty rice weevils of each strain were collected independently three times and immediately frozen at −70°C for total RNA extraction. The frozen rice weevils were rinsed twice with the DEPC-containing water and homogenized using a pencil-type homogenizer in 1 mL Trizol reagent. The homogenates were incubated on ice for 20 min, and total RNA was extracted according to the manufacturer’s protocol. The quality of total RNA was determined by measuring the absorbance at 260 and 280 nm (A_260/280_) and by agarose gel electrophoresis. Subsequently, cDNA was synthesized using the Maxima First Strand cDNA Synthesis Kit (Thermo Fisher Scientific Inc., Waltham, MA) and was stored at −20°C until use. The qPCR was performed on a QuantStudio 3 Real-Time PCR System (Applied biosystems, Foster City, CA) using Luna® Universal qPCR Master Mix (New England Biolabs, Ipswich, MA), according to the manufacturer’s instructions. All qPCRs were performed in duplicate. Primers were designed by aligning the protein and mRNA sequences of the top five species of arthropods that displayed alignment with annotated proteins from the proteomics data. Multiple sequence alignments were conducted using MAFFT version 7 [3] for the identification of conserved regions. All primers were designed in the most conserved regions, and both ends of each primer contained more than five nucleotides conserved in the top five species (**Additional file 1: Table S4**). Transcript levels of genes were normalized relative to two reference genes including those encoding ribosomal protein L29 (*rpl29*) and glyceraldehyde-3-phosphate dehydrogenase (*gapdh*) [4]. The results of qRT-PCR were calculated using the 2^-∆∆Ct^ method [5]. Heat map was constructed using Log_2_ gene expression ratio between the WT and the PH_3_–R strains.

**2. Supplementary Tables**

**Table S1. Alignment of dihydrolipoamide dehydrogenase (DLD) of PH_3_ susceptible (WT) and resistant (R1 and R2) strains.**

| **Strains** | **DLD (492–506 amino acids)^a^** | | | | | | | | | | | | | | | **Detection of**  **SNP^b^** |
| --- | --- | --- | --- | --- | --- | --- | --- | --- | --- | --- | --- | --- | --- | --- | --- | --- |
| WT | R | E | A | N | V | S | A | A | F | G | K | P | I | N | F | No |
| R1 | R | E | A | N | V | S | A | A | F | G | K | P | I | N | F | No |
| R2 | R | E | A | N | V | S | A | A | F | G | K | P | I | T | F | Yes |

**^a^**DLD amino acid sequence (accession number :ALY05704.1). The mutation is highlighted in gray.

**^b^**SNP, single nucleotide polymorphism. SNP in the *dld* gene was detected as described previously [1].

**Table S2. Kinetic parameters of COX in WT, MR, and SR strains upon exposure to ethyl formate.**

| **Strains** | **Values**^†^ | **Ethyl formate concentrations** | | |
| --- | --- | --- | --- | --- |
|  |  | **0 mM** | **1 mM** | **10 mM** |
| **WT** | K_i_ | 26.479 ± 2.333 | | |
|  | V_max_ | 15.579 ± 2.478 | 15.045 ± 1.687 | 6.853 ± 0.300 |
| **MR** | K_i_ | 10.200 ± 0.843 | | |
|  | V_max_ | 6.120 ± 0.789 | 6.180 ± 0.353 | 2.977 ± 0.301 |
| **SR** | K_i_ | 8.544 ± 2.504 | | |
|  | V_max_ | 3.270 ± 0.650 | 2.216 ± 0.716 | 2.636 ± 1.284 |

^†^The inhibition constant (K_i_) and V_max_ were calculated using a model of enzyme kinetics-inhibition with noncompetitive inhibition mode for the PH_3_ susceptible (WT) and both PH_3_–R strains (moderately resistant [MR] and strongly resistant [SR]) using GraphPad Prism version 8.0.1 (La Jolla, CA).

**Table S3. Fold-change in protein levels in WT and PH_3_-R strains using nLC-ESI-MS/MS**

| **Accession No.** | **Identified Proteins** | **Abbreviation** | **Log_2_ (Fold)** | **-Log_10_(P-value)** |
| --- | --- | --- | --- | --- |
| 1. **SR vs. WT** | | | | |
| EEZ98544.2 | Myosin light chain alkali-like Protein [*T. castaneum*] | MLC1 | 4.13 | 1.18 |
| pir\|\|JC5771 | chaperonin groEL-like protein - Weevil | groEL | 3.09 | 11.16 |
| EFA09221.1 | NADH-ubiquinone oxidoreductase 75 kDa subunit, mitochondrial-like Protein [*T. castaneum*] | ND | 1.76 | 1.72 |
| EFA10458.1 | Troponin C, isoform 2-like Protein [*T. castaneum*] | TnC2 | 1.61 | 3.67 |
| EFA09970.1 | 40S ribosomal protein S15Aa-like Protein [*T. castaneum*] | RPS15 | 1.41 | 1.62 |
| AHF20221.1 | heat shock protein 90 [*T. castaneum*] | Hsp90 | 1.13 | 1.19 |
| EFA10538.1 | GTP-binding nuclear protein Ran-like Protein [*T. castaneum*] | Ran | 1.07 | 1.38 |
| EFA02331.1 | 26S protease regulatory subunit 4-like Protein [*T. castaneum*] | PSMC1 | 1.04 | 1.06 |
| KYB24970.1 | Tropomyosin-2-like Protein [*T. castaneum*] | TPM2 | 1.00 | 1.82 |
| EFA01051.1 | 60S ribosomal protein L14-like Protein [*T. castaneum*] | RPL14 | 0.93 | 1.68 |
| EFA06522.1 | Selenide, water dikinase-like Protein [*T. castaneum*] | SEPH | 0.79 | 1.63 |
| EFA11293.1 | 40S ribosomal protein S6-like Protein [*T. castaneum*] | N/A | 0.73 | 1.03 |
| EEZ99233.1 | alpha spectrin [*T. castaneum*] | a-spectrin | 0.64 | 1.54 |
| CAY35674.1 | unnamed protein product [*T. castaneum*] Crotonase-like | Crotonase-like | -4.79 | 1.68 |
| EFA01574.1 | Muscle-specific protein 20-like Protein [*T. castaneum*] | MSP20 | -3.70 | 2.47 |
| EEZ99521.1 | Cluster of ADP,ATP carrier protein-like Protein [*T. castaneum*] | AAC | -3.15 | 2.00 |
| EEZ99658.1 | ADP,ATP carrier protein-like Protein [*T. castaneum*] | AAC | -2.82 | 2.48 |
| KYB27628.1 | Profilin-like Protein [*T. castaneum*] | PFN | -2.57 | 1.60 |
| EFA06341.1 | 40S ribosomal protein S7-like Protein [*T. castaneum*] | RSP7 | -2.50 | 1.44 |
| EEZ99469.1 | cAMP-dependent protein kinase type I regulatory subunit-like Protein [*T. castaneum*] | PRKAR1A | -2.35 | 2.47 |
| EEZ99803.1 | Histone H2A-like Protein [*T. castaneum*] | N/A | -2.31 | 1.19 |
| EFA09609.1 | Enolase-like Protein [*T. castaneum* ] | Enolase-like | -2.20 | 1.12 |
| EFA08118.1 | Actin-interacting protein 1-like Protein [*T. castaneum*] | ACP1 | -2.06 | 2.21 |
| EFA08228.2 | 6-phosphofructokinase-like Protein [*T. castaneum* | PFK1 | -2.04 | 1.56 |
| EFA04932.1 | Fructose-bisphosphate aldolase-like Protein [*T. castaneum*] | FBA | -2.01 | 3.11 |
| ADU33252.1 | glycoside hydrolase family protein 48 [*S. oryzae*] | GH | -1.99 | 2.29 |
| ADU33259.1 | pectin methylesterase [*S. oryzae*] | PME | -1.76 | 3.18 |
| EFA01748.1 | Triosephosphate isomerase-like Protein [*T. castaneum*] | N/A | -1.69 | 1.02 |
| CON__P01966 | CON__P01966 | N/A | -1.67 | 1.47 |
| ADU33251.1 | Cluster of glycoside hydrolase family protein 48 [*S. oryzae*] | GH | -1.62 | 3.05 |
| EFA08121.1 | Aconitate hydratase, mitochondrial-like Protein [*T. castaneum*] | ACON | -1.61 | 1.23 |
| CBL74949.1 | unnamed protein product [*T. castaneum*] Adenylate kinase-like | AK-like | -1.56 | 1.39 |
| EFA07687.2 | Glucose-6-phosphate isomerase-like Protein [*T. castaneum*] | GPI | -1.45 | 3.12 |
| CBM37118.1 | unnamed protein product [*T. castaneum*] ATP-dependent 26S-proteasome regulatory subunit | 26S PR | -1.42 | 1.19 |
| EFA05271.2 | putative medium-chain specific acyl-CoA dehydrogenase, mitochondrial-like Protein [*T. castaneum*] | MCAD | -1.40 | 2.57 |
| AMR73093.1 | Cluster of arginine kinase, partial [*Sitophilus* sp. 1 SM-2016] | AK | -1.37 | 1.18 |
| ABH88184.1 | chemosensory protein 11 [*T. castaneum*] | CSP11 | -1.33 | 3.37 |
| ALM55745.1 | thaumatin-like protein [*S. oryzae*] | Thaumatin | -1.31 | 1.56 |
| EFA05014.1 | ATP-citrate synthase-like Protein [*T. castaneum*] | ACS | -1.13 | 4.01 |
| ADM73187.1 | alpha-amylase [*S. oryzae*] | AA | -1.07 | 3.13 |
| EEZ99726.1 | Nucleoside diphosphate kinase-like Protein [*T. castaneum*] | NDK | -1.06 | 2.18 |
| AHY84716.1 | clathrin heavy chain [*T. castaneum*] | Clathrin heavy chain | -0.96 | 2.04 |
| EFA03596.1 | 60 kDa heat shock protein, mitochondrial-like Protein [*T. castaneum*] | Hsp60 | -0.90 | 1.33 |
| AAN08765.1 | Cluster of elongation factor 1-alpha, partial [*S. oryzae*] | EEF1A1 | -0.87 | 2.09 |
| EFA05436.2 | Acetyl-CoA carboxylase-like Protein [*T. castaneum*] | ACC | -0.82 | 3.48 |
| AAV40982.1 | heat shock protein 70, partial [*T. castaneum*] | Hsp70 | -0.77 | 1.65 |
| EFA01731.1 | 60S ribosomal protein L10a-2-like Protein [*T. castaneum*] | RPL10 | -0.74 | 1.28 |
| ADU33246.1 | endo-beta-1,4-glucanase [*S. oryzae*] | EG | -0.73 | 3.11 |
| EFA08517.1 | Glyceraldehyde-3-phosphate dehydrogenase 2-like Protein [*T. castaneum*] | GAPDH2 | -0.63 | 2.18 |
| ADU33262.1 | pectin methylesterase [*S. oryzae*] | PME | -0.61 | 1.72 |
| EFA10733.1 | Peroxiredoxin 1-like Protein [*T. castaneum*] | N/A | -0.61 | 1.06 |
| EFA05965.1 | Elongation factor 2-like Protein [*T. castaneum*] | N/A | -0.60 | 1.17 |
| 1. **MR vs. WT** | | | | |
| CON__P01966 | CON__P01966 | N/A | 3.58 | 1.96 |
| pir\|\|JC5771 | chaperonin groEL-like protein - Weevil | groEL | 3.21 | 9.73 |
| EFA09970.1 | 40S ribosomal protein S15Aa-like Protein [Tribolium castaneum] | RPS15 | 1.99 | 3.82 |
| KYB24981.1 | Heterogeneous nuclear ribonucleoprotein 27C-like Protein [*T. castaneum*] | HRB27C | 1.81 | 1.78 |
| EFA08798.1 | 26S protease regulatory subunit 8-like Protein [*T. castaneum*] | PSMC5 | 1.70 | 1.38 |
| EFA02331.1 | 26S protease regulatory subunit 4-like Protein [*T. castaneum*] | PSMC1 | 1.60 | 2.76 |
| CAI45288.1 | phosphatase [*T. castaneum*] | Phosphatase | 1.56 | 2.59 |
| EFA10538.1 | GTP-binding nuclear protein Ran-like Protein [*T. castaneum*] | Ran | 1.46 | 2.18 |
| CON__P02538 | CON__P02538 | N/A | 1.45 | 1.10 |
| EFA06522.1 | Selenide, water dikinase-like Protein [*T. castaneum*] | SEPH | 1.40 | 2.09 |
| EEZ97876.1 | Proteasome subunit alpha type-1-like Protein [*T. castaneum*] | PSMA1 | 1.37 | 1.56 |
| EEZ99587.1 | Cofilin/actin-depolymerizing factor homolog-like Protein [*T. castaneum*] | N/A | 1.24 | 1.04 |
| XP_008200725.1 | PREDICTED: LOW QUALITY PROTEIN: 14-3-3 protein zeta [*T. castaneum*] | 14-3-3 | 1.14 | 1.12 |
| CBM37119.1 | unnamed protein product [*T. castaneum*] ATP-dependent 26S proteasome regulatory | 26S PR | 1.10 | 1.84 |
| EFA08461.1 | 14-3-3 protein epsilon-like Protein [*T. castaneum*] | 14-3-3 | 1.09 | 1.10 |
| EEZ98819.2 | Heat shock 70 kDa protein cognate 3-like Protein [Tribolium castaneum] | N/A | 1.02 | 1.04 |
| EFA04313.1 | hypothetical protein TcasGA2_TC014606 [*T. castaneum*] | Hsp83-like | 0.94 | 1.47 |
| KYB24621.1 | Stress-induced-phosphoprotein 1-like Protein [*T. castaneum*] | STIP1 | 0.91 | 1.35 |
| EFA03441.1 | 40S ribosomal protein S4-like Protein [*T. castaneum*] | RPS4 | 0.91 | 1.72 |
| EFA09061.1 | Guanine nucleotide-binding protein subunit beta-like protein [*T. castaneum*] | N/A | 0.81 | 1.68 |
| EFA01887.1 | Rab proteins geranylgeranyltransferase component A-like Protein [*T. castaneum*] | GGTase | 0.80 | 1.68 |
| KYB25938.1 | G protein-coupled receptor kinase 1-like Protein [*T. castaneum*] | N/A | 0.77 | 1.12 |
| EFA09539.1 | Isocitrate dehydrogenase [NADP] cytoplasmic-like Protein [*T. castaneum*] | IDH | 0.77 | 1.76 |
| EFA01574.1 | Muscle-specific protein 20-like Protein [*T. castaneum*] | MSP20 | -4.14 | 2.47 |
| CAY35674.1 | unnamed protein product [*T. castaneum*] Crotonase-like | Crotonase-like | -3.86 | 1.68 |
| EEZ99658.1 | ADP,ATP carrier protein-like Protein [*T. castaneum*] | AAC | -3.70 | 3.11 |
| EFA06341.1 | 40S ribosomal protein S7-like Protein [*T. castaneum*] | RSP7 | -2.77 | 1.44 |
| EEZ98030.1 | Muscle-specific protein 20-like Protein [*T. castaneum*] | MSP20 | -2.26 | 1.06 |
| EFA05271.2 | putative medium-chain specific acyl-CoA dehydrogenase, mitochondrial-like Protein [*T. castaneum*] | MCAD | -2.12 | 1.81 |
| EFA07448.1 | ATP synthase subunit gamma, mitochondrial-like Protein [*T. castaneum*] | ATP5F1C | -2.11 | 1.38 |
| EFA08228.2 | 6-phosphofructokinase-like Protein [*T. castaneum*] | PFK1 | -2.04 | 1.28 |
| EFA10456.1 | 4-hydroxyphenylpyruvate dioxygenase-like Protein [*T. castaneum*] | HPPD | -1.99 | 2.18 |
| ADU33251.1 | Cluster of glycoside hydrolase family protein 48 [Sitophilus oryzae] | GH | -1.47 | 3.42 |
| EFA08118.1 | Actin-interacting protein 1-like Protein [*T. castaneum*] | ACP1 | -1.46 | 1.75 |
| EFA07428.1 | ATP synthase subunit alpha, mitochondrial-like Protein [Tribolium castaneum] | ATP5F1A | -1.45 | 2.33 |
| EEZ99726.1 | Nucleoside diphosphate kinase-like Protein [*T. castaneum*] | NDK | -1.41 | 2.18 |
| EFA09226.1 | Succinate dehydrogenase [ubiquinone] flavoprotein subunit, mitochondrial-like Protein [*T. castaneum*] | SDH | -1.37 | 1.35 |
| ADM73187.1 | alpha-amylase [Sitophilus oryzae] | AA | -1.32 | 3.25 |
| EEZ99469.1 | cAMP-dependent protein kinase type I regulatory subunit-like Protein [*T. castaneum*] | PRKAR1A | -1.31 | 2.71 |
| EFA07147.1 | Ras-related protein Rab-26-like Protein [*T. castaneum*] | Rab26 | -1.18 | 1.96 |
| ANW06522.1 | cytochrome c oxidase subunit II (mitochondrion) [Sitophilus oryzae] | COX2 | -1.04 | 1.55 |
| ADU33252.1 | glycoside hydrolase family protein 48 [*S. oryzae*] | GH | -1.03 | 1.55 |
| EEZ98942.2 | Inorganic pyrophosphatase-like Protein [*T. castaneum*] | N/A | -0.96 | 1.15 |
| EFA10430.1 | Calcium-transporting ATPase sarcoplasmic/endoplasmic reticulum type-like Protein [*T. castaneum*] | SERCA | -0.69 | 2.72 |
| EFA09609.1 | Enolase-like Protein [*T. castaneum*] | Enolase-like | -0.68 | 2.83 |
| EFA05714.1 | ATP synthase subunit beta, mitochondrial-like Protein [*T. castaneum*] | ATP5F1B | -0.63 | 1.73 |
| EFA05476.1 | Protein l(2)37Cc-like Protein [*T. castaneum*] | I(2)37Cc | -0.62 | 1.06 |

The proteins are sorted according to 1.5 fold-change in each PH_3_-R strain versus WT. Only the proteins having less than 0.1 *p*-values are shown.

**Table S4. List of qRT-PCR primers used in this study**

| **Gene name** | **Abbreviation** | **Primer Sequence (5′→3′)** | | **Size (bp)** |
| --- | --- | --- | --- | --- |
| Glyceraldehyde 3-phosphate dehydrogenase | *gapdh* | F | AACTTTGCCGACAGCCTTGG | 277 |
|  |  | R | GCGCCCATGTATGTAGTTGG |  |
| Libosomal protein L29 | *lpl29* | F | TGGCCAAGTCCAAGAATCACA | 250 |
|  |  | R | TTCTTGGCGCTAGCTTGTCTT |  |
| Glucose-6-phosphate isomerase | *gpi* | F | ATGTTTGGATTTTGGGATTGGGT | 287 |
|  |  | R | TCCCACTTATCCAGATTTTCCAGGAT |  |
| Fructose-bisphosphate aldolase | *fba* | F | GAAGGCACTCTCTTGAAGCCCAACATG | 251 |
|  |  | R | GCTTGGAGAGCACGTCCATAACTGAA |  |
| ATP synthase subunit α, mitochondrial | *atp5f1a* | F | ATTCCCACCAATGTAATTTCTATCAC | 143 |
|  |  | R | TGTTTCATGGCCTTGGTTTG |  |
| ATP synthase subunit β, mitochondrial | *atp5f1b* | F | GAAATGATTGAGTCCGGTGTCATCTC | 85 |
|  |  | R | GCCTGGTGGTTCGTTCATCTG |  |
| cAMP-dependent protein kinase regulatory subunit | *prkar1a* | F | TGGGGCATTGACCGAGACTC | 119 |
|  |  | R | TCCCACTTATCCAGATTTTCCAGGAT |  |
| ADP,ATP carrier protein 1 | *aac1* | F | AAGGGAATGTTGCCCGATC | 131 |
|  |  | R | GACTGCATCATCATACGCCT |  |
| Profilin | *pfn* | F | ATGAGCTGGCAGGATTACGT | 98 |
|  |  | R | GATTTGGCCCAGACATTTCCATC |  |
| Alpha-amylase | *amy1* | F | AAGCGTTGTCGTAGAAGGCA | 90 |
|  |  | R | AAGGCTGCTTCATCTCCACC |  |
| Glycoside hydrolase family protein 48 | *gh48* | F | GGAGACCCACCAAATGCTCA | 107 |
|  |  | R | TCGTTGGCTTTAGCAGCGTA |  |
| Cytochrome oxidase subunit II | *cox2* | F | TCGACTCTTAGTTACGTCTGC | 85 |
|  |  | R | GCGTCCTGGGGTTCTATCAA |  |
| Pectin methylesterase | *pme* | F | ATGGACAAGGGCAGATCACC | 109 |
|  |  | R | ACCCCAGTCACCGAAAACAG |  |
| Dihydrolipoamide dehydrogenase E3 subunit | *dld* | F | TGGGGCTGGTGTTATTGGTG | 109 |
|  |  | R | TTGGTCGATACCTACGCCAC |  |
| NADH-ubiquinone oxidoreductase 75 kDa subunit | *ndufs1* | F | ATGCCTGTTATGAAAGGGTGG | 122 |
|  |  | R | CCTTGGTCACAAATTGGGCAAT |  |
| Rab proteins geranylgeranyltransferase component A | *chm* | F | ATGTATGGATTTGGAGAACTGCCACAAGG | 170 |
|  |  | R | TCGCAGTAGACCTGCTTGCATTT |  |
| Heat shock protein 90 | *hsp90* | F | TTCCAGAACGTGGCCAAGGA | 215 |
|  |  | R | TCCATGTTTCCGGTCCAGCC |  |
| Tropomyosin-2 | *tpm2* | F | CAGAAGGAAGTTGACAGGCTTGAAGA | 93 |
|  |  | R | TTCGGCGAAGGTGGAGTCC |  |
| Myosin heavy chain 1 | *myh1* | F | AAGCCCAGGATCAAGGTCG | 181 |
|  |  | R | CCAGTACACCAATCAAGTGCTG |  |
| Actin, muscle | *acta2* | F | GGTATTCTCACTTTGAAATACCCCAT | 86 |
|  |  | R | TAGAGGTGTGATGCCAGATCTT |  |

**Table S5. Mitochondrial genetic code of *S. oryzae.***

|  |  | **Second Base** | | | |  |  |
| --- | --- | --- | --- | --- | --- | --- | --- |
|  |  | **U** | **C** | **A** | **G** |  |  |
| **First**  **Base** | **U** | Phe | Ser | Tyr | Cys | **U** | **Third**  **Base** |
|  |  | Phe | Ser | Tyr | Cys | **C** |  |
|  |  | Leu | Ser | Stop | Trp | **A** |  |
|  |  | Leu | Ser | Stop | Trp | **G** |  |
|  | **C** | Leu | Pro | His | Arg | **U** |  |
|  |  | Leu | Pro | His | Arg | **C** |  |
|  |  | Leu | Pro | Gln | Arg | **A** |  |
|  |  | Leu | Pro | Gln | Arg | **G** |  |
|  | **A** | Ile | Thr | Asn | Ser | **U** |  |
|  |  | Ile | Thr | Asn | Ser | **C** |  |
|  |  | Met | Thr | Lys | Ser | **A** |  |
|  |  | Met | Thr | Lys | Ser | **G** |  |
|  | **G** | Val | Ala | Asp | Gly | **U** |  |
|  |  | Val | Ala | Asp | Gly | **C** |  |
|  |  | Val | Ala | Glu | Gly | **A** |  |
|  |  | Val | Ala | Glu | Gly | **G** |  |

**3. Supplementary Figures**

| ***cox1*** |  |  |  |
| --- | --- | --- | --- |
| **1407-** | S | GGA ATA GTA GGT ACA TC**C** TTA AGT TTG CTA ATT CGG GCA GAA | **-1448** |
|  | MR | GGA ATA GTA GGT ACA TC**C** TTA AGT TTG CTA ATT CGG GCA GAA |  |
|  | SR | GGA ATA GTA GGT ACA TC**T** TTA AGT TTG CTA ATT CGG GCA GAA |  |
|  |  | **S > S** |  |
| **1707-** | S | ACC GTC TAC CCC CCG CTC TCA TC**T** AAT ATT GCC CAT GAA GGA | **-1748** |
|  | MR | ACC GTC TAC CCC CCG CTC TCA TC**T** AAT ATT GCC CAT GAA GGA |  |
|  | SR | ACC GTC TAC CCC CCG CTC TCA TC**C** AAT ATT GCC CAT GAA GGA |  |
|  |  | **S > S** |  |
| ***cox2*** |  |  |  |
| **3187-** | S | TTA TAC ATT CTA GA**C** GAA ATT AAT AAC CCT TCT ATT ACT ATT | **-3228** |
|  | MR | TTA TAC ATT CTA GA**C** GAA ATT AAT AAC CCT TCT ATT ACT ATT |  |
|  | SR | TTA TAC ATT CTA GA**T** GAA ATT AAT AAC CCT TCT ATT ACT ATT |  |
|  |  | **D** > **D** |  |
| ***nad1*** |  |  |  |
| **12303-** | S | ATT TGT GTT TTA GTT GGG GTT GGA TTT CT**G** ACT TTA ATA GAG | **-12344** |
|  | MR | ATT TGT GTT TTA GTT GGG GTT GGA TTT CT**G** ACT TTA ATA GAG |  |
|  | SR | ATT TGT GTT TTA GTT GGG GTT GGA TTT CT**A** ACT TTA ATA GAG |  |
|  |  | **L** > **L** |  |
| ***nad2*** |  |  |  |
| **1088-** | S | AAC TCT ATT AAC TTT ACA GG**A** CTA ATT TTA ACC TCA ATT TTT | **-1129** |
|  | MR | AAC TCT ATT AAC TTT ACA GG**A** CTA ATT TTA ACC TCA ATT TTT |  |
|  | SR | AAC TCT ATT AAC TTT ACA GG**G** CTA ATT TTA ACC TCA ATT TTT |  |
|  |  | **G** > **G** |  |
| ***nad4*** |  |  |  |
| **9066-** | S | TTT TGA ATT TGC TCA TTA ATA TTA TTA G**C**A AGA GCA AAA ATT | **-9107** |
|  | MR | TTT TGA ATT TGC TCA TTA ATA TTA TTA G**C**A AGA GCA AAA ATT |  |
|  | SR | TTT TGA ATT TGC TCA TTA ATA TTA TTA G**A**A AGA GCA AAA ATT |  |
|  |  | **A** > **E** |  |
| **8262-** | S | GGT TTA ATG AAT TTT AT**G** CCA ACA TTA TCT ATA TGA TGG TTT | **-8303** |
|  | MR | GGT TTA ATG AAT TTT AT**G** CCA ACA TTA TCT ATA TGA TGG TTT |  |
|  | SR | GGT TTA ATG AAT TTT AT**A** CCA ACA TTA TCT ATA TGA TGG TTT |  |
|  |  | **M** > **M** |  |
| **8202-** | S | ATA GCC GCT CCT CCT TCA TTA AAT TTG TT**G** GGG GAA GTC CTT | **-8243** |
|  | MR | ATA GCC GCT CCT CCT TCA TTA AAT TTG TT**G** GGG GAA GTC CTT |  |
|  | SR | ATA GCC GCT CCT CCT TCA TTA AAT TTG TT**A** GGG GAA GTC CTT |  |
|  |  | **L** > **L** |  |
| ***Nad4*** | | |  |
| **8076-** | S | TTT TTA TAT TCT TAT ACT CAG CAT GG**T** TTA TTT AGT TCT GGA | **-8117** |
|  | MR | TTT TTA TAT TCT TAT ACT CAG CAT GG**T** TTA TTT AGT TCT GGA |  |
|  | SR | TTT TTA TAT TCT TAT ACT CAG CAT GG**G** TTA TTT AGT TCT GGA |  |
|  |  | **G** > **G** |  |
| **Nad4l** |  |  |  |
| **9290-** | S | CAT GGG AAT GAT TAT GTA **C**TA TCT TTT TCT TCT TTA TGA TAA | **-9331** |
|  | MR | CAT GGG AAT GAT TAT GTA **C**TA TCT TTT TCT TCT TTA TGA TAA |  |
|  | SR | CAT GGG AAT GAT TAT GTA **T**TA TCT TTT TCT TCT TTA TGA TAA |  |
|  |  | **L** > **L** |  |
| ***nad5*** |  |  |  |
| **7527-** | S | ATT TTA TTA GGT TGA GAC GGA TTA GGG **C**TA ATT TCT TAT ATT | **-7568** |
|  | MR | ATT TTA TTA GGT TGA GAC GGA TTA GGG **C**TA ATT TCT TAT ATT |  |
|  | SR | ATT TTA TTA GGT TGA GAC GGA TTA GGG **T**TA ATT TCT TAT ATT |  |
|  |  | **L** > **L** |  |
| **7386-** | S | TAT TTT TTA GAA TAT GGA AGT TGA AAC TTT A**G**T AGA TTC TTA | **-7426** |
|  | MR | TAT TTT TTA GAA TAT GGA AGT TGA AAC TTT A**G**T AGA TTC TTA |  |
|  | SR | TAT TTT TTA GAA TAT GGA AGT TGA AAC TTT A**A**T AGA TTC TTA |  |
|  |  | **S** > **N** |  |
| **7344-** | S | GA**C** TTT TAT AAT TTA AAA GAG AAT ATG GGT TTA ACA CTA ATT | **-7385** |
|  | MR | GA**C** TTT TAT AAT TTA AAA GAG AAT ATG GGT TTA ACA CTA ATT |  |
|  | SR | GA**G** TTT TAT AAT TTA AAA GAG AAT ATG GGT TTA ACA CTA ATT |  |
|  |  | **D** > **E** |  |
| **6648-** | S | TTT AAT TTT ATT AGA TTG AAT TCA ATT GG**C** GAT AAA AGA GAA | **-6689** |
|  | MR | TTT AAT TTT ATT AGA TTG AAT TCA ATT GG**C** GAT AAA AGA GAA |  |
|  | SR | TTT AAT TTT ATT AGA TTG AAT TCA ATT GG**T** GAT AAA AGA GAA |  |
|  |  | **G** > **G** |  |
| ***nad6*** |  |  |  |
| **10017-** | S | A**A**T ATC TTA AAT AAA AAT TAT AGA ACC TTT ATC CAA TTT AAC | **-10058** |
|  | MR | A**A**T ATC TTA AAT AAA AAT TAT AGA ACC TTT ATC CAA TTT AAC |  |
|  | SR | A**G**T ATC TTA AAT AAA AAT TAT AGA ACC TTT ATC CAA TTT AAC |  |
|  |  | **N** > **S** |  |
| ***atp6*** |  |  |  |
| **3926-** | S | TCT TCC TTT GAC CCA TCA ACT AA**T** TTT AAC TCA ACT TTA AAC | **-3967** |
|  | MR | TCT TCC TTT GAC CCA TCA ACT AA**T** TTT AAC TCA ACT TTA AAC |  |
|  | SR | TCT TCC TTT GAC CCA TCA ACT AA**C** TTT AAC TCA ACT TTA AAC |  |
|  |  | **N** > **N** |  |

**Fig. S1. Mitochondrial gene sequences carrying point mutations in the S, MR, and SR strains.**

**
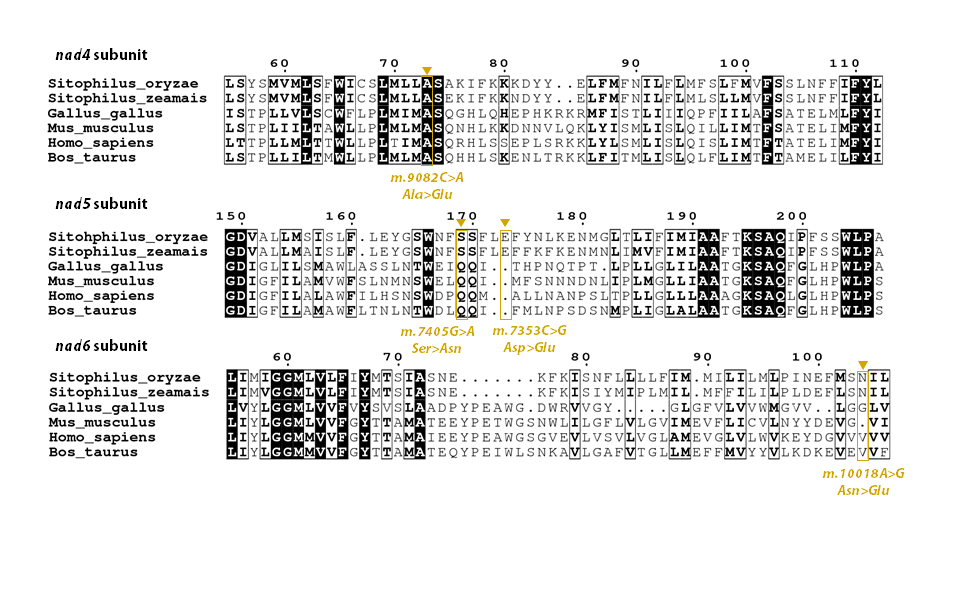
**

**Fig. S2. Alignments of deduced amino acid sequences of mtDNA sequences encoding ND subunits with point mutations.** *Sitophilus oryzae* (National Center for Biotechnology Information protein database accession no. for nad4, nad5, and nad6 subunits: ANW06528.1, ANW06527.1, and ANW06530.1, respectively); *Sitophilus zeamais* (YP_009271136.1, YP_009271135.1, YP_009271138.1); *Gallus gallus* (NP_006924.2, NP_006925.1, NP_006927.1); *Mus musculus* (NP_904337.1, NP_904338.1, NP_904339.1); *Homo sapiens* (YP_003024035.1, YP_003024036.1, YP_003024037.1), *Bos taurus* (YP_209214.1, YP_209215.1, YP_209216.1).

**References**

1. Nguyen TT, Collins PJ, Duong TM, Schlipalius DI, Ebert PR: **Genetic conservation of phosphine resistance in the rice weevil *Sitophilus oryzae* (L.)**. *J Hered* 2016, **107**(3):228-237.

2. Bliss CI: **The Method of Probits**. *Science* 1934, **79**(2037):38-39.

3. Standley DM, Katoh K: **MAFFT Multiple Sequence Alignment Software Version 7: Improvements in Performance and Usability**. *Molecular Biology and Evolution* 2013, **30**(4):772-780.

4. Vigneron A, Charif D, Vincent-Monégat C, Vallier A, Gavory F, Wincker P, Heddi AJBM: **Host gene response to endosymbiont and pathogen in the cereal weevil Sitophilus oryzae**. 2012, **12**(1):S14.

5. Livak KJ, Schmittgen TD: **Analysis of relative gene expression data using real-time quantitative PCR and the 2−ΔΔC_T_ method**. *Methods* 2001, **25**(4):402-408.
